# Supplementary material for: Nutritional assessment of adolescents: A cross-sectional study from public schools of North India
Source: PLoS One. 2025 Jan 14;20(1):e0316435. doi: 10.1371/journal.pone.0316435 (PMC11731870; doi:10.1371/journal.pone.0316435)
Supplement: S1 File — (PDF) [file pone.0316435.s002.pdf]

Supplement

Nutritional Assessment of Adolescents: a Cross-sectional Study from Public Schools of North India

CONTENTS

APPENDIX S1: 24-Hour Urine Collection: Field-Form 1

APPENDIX S2 2

APPENDIX S2-1: Standard Operating Procedure 2

APPENDIX S2-2: Laboratory Methods 4

APPENDIX S3 5

APPENDIX S3-1: List of Food Items in Pure Study Software 5

APPENDIX S3-2: Micro-Nutrients, Vitamins and Minerals Estimation Using Pure Study Software 7

APPENDIX S4 8

APPENDIX S4-1: Salt and Sugar Estimation 8

APPENDIX S4-2: Fruits and Vegetable Estimation 9

APPENDIX S5 10

APPENDIX S5-1: Participant Informed Assent Form 10

APPENDIX S5-2: Parental Informed Consent Form 11

APPENDIX S6: Number of Adolescent Participants for Each Indicator (%) 13

APPENDIX S7 14

TABLE S1: Socio-demographic Characteristics of the Participating Adolescents (n=453) 14

APPENDIX S8 15

Table S2: Dietary Intake among Adolescents (n=453) 15

Table S3: Urinary Excretory Salt Levels among the Adolescents 16

Table S4: Anthropometric Measures among Adolescents 17

APPENDIX S9 18

Table S5: Recommended Dietary Allowances of Macronutrients among Adolescents 18

Table S6: Recommended Dietary Allowances of Minerals among Adolescents 19

Table S7: Recommended Dietary Allowances of Vitamins among Adolescents 20

APPENDIX S10 21

Table S8: Percentage energy intake from carbohydrates and fats among Adolescents 21

APPENDIX S1: 24-Hour Urine Collection: Field-Form

Please fill the following information:

Name:
Age:

Code:

Collection	Start Date	Start Time	Collected/Missed
Collection 1			
Collection 2			
Collection 3			
Collection 4			
Collection 5			
Collection 6			
Collection	Stop Date	Stop Time	

Signature with date:

APPENDIX S2

APPENDIX S2-1: Standard Operating Procedure

Introduction: A 24-hour collection of urine into a plain container can be used to estimate excretion of sodium.

Purpose: The SOP provides instructions for specimen collection, handling and labeling to promote uniformity.

Policy: All specimens must be labeled with the participants name and a special six digit code already assigned to each participant. For parents 8 digit codes that include age will be used.

Equipment (for one participant)
Participant information sheet
One 3-litre 24hr urine collection container. No additives.
Jug
two 50 ml container for final sample collection of 24-hour urine sample sample

Procedure: Pre-preparation
Labeling Specimens and Containers
Information must be placed on a label which is on the container.
All specimens MUST be labeled with the participants name and a special six digit code already assigned to each participant. For parents 8 digit codes that include age will be used.
Identifying information placed on covers of containers is NOT acceptable.

Outside Contamination of Specimen Container
The outside of containers or accompanying requisition slips should not be contaminated, (i.e. contamination of a container with feces, urine, etc.)
If the specimen container is inadvertently contaminated at the time of collection and it is not feasible to recollect a specimen, the contaminated container should be placed inside a plastic bag and sealed, and this fact should be noted on the requisition slip.
Contaminated requisition slips should be discarded and a new one filled out.

Damaged Specimen Container
Glass or plastic containers which are cracked or broken so that the specimen may leak out (or may be considered a hazard to those handling the object) should not be submitted for examination. Dispose in the appropriate rigid, biohazard container.
Specimens must be delivered promptly to the laboratory after collection.

Steps for sample collection
Each participant will be visited by a research team member.
It is important that the participant understand the process, and to that end, a full verbal explanation should be given along with a written information sheet repeating the same details.
This sheet should have a contact name and phone number included, allowing the patient to call and ask any questions or clarify any doubts.
Participants will be given schedule sheet where they will note down the time each time they pass urine and will also note time and mention if they fail to collect any sample during the 24 hour time.
Ensure that the participant understands that even a single sample collection missed will impair/invalidate the results obtained from that collection.
Following instructions to be shared with participants for 24-Hour Urine Collection

Instruct the participant to avoid alcoholic beverages, vitamins, and other medication (if possible) after they have been provided with the containers for sample collection till the time the samples are collected back from them.
Instruct the participant to check with his/her physician prior to discontinuing any medications.
Instruct the participant to not exceed his/her normal intake of liquids during the day before and the day of collection unless his/her physician gives the participant specific directions to do otherwise.
Instruct the participant to keep the specimen in cool & dark place during the 24 hour collection period.

The 24-hour collection period begins when the participant gets up in the morning and empties his/her bladder. DO NOT COLLECT THIS URINE! But do record the date and time of this voiding on the Sheet provided. Be sure to collect ALL urine (Day and Night) for the next 24 hours.

Instruct the participant to make his/her final collection when he/she empties his/her bladder the next morning, approximately 24 hours from the time marked on his/her specimen container.

APPENDIX S2-2: Laboratory Methods

All urine samples were analyzes in Beckman Coulter AU 5800 analyzer (2020)

Principle

ISE Electrolyte Buffer reagent and ISE Electrolyte Reference reagent, when used in conjunction with UniCel DxC 600/800 System(s) and SYNCHRON Systems AQUA CAL 1, 2 and 3, are intended for the quantitative determination of sodium concentration in human serum, plasma or urine.

Methodology

The SYNCHRON System(s) determines sodium ion concentration by indirect potentiometry utilizing two glass sodium electrodes (one acts as the reference electrode). To measure sodium concentrations, a precise volume of sample (40 microliters) is mixed with a buffered solution. The ratio used is one part sample to 33 parts buffer. The high molar strength buffer is used to establish a constant activity coefficient for sodium ions, calibrating the electrode to concentration values.

Chemical Reaction Scheme

The sodium electrode is made of lithium-sodium-aluminum-silicate glass. It is essential that the outer layer of the glass electrode is adequately hydrated. When the sample buffer mixture contacts the electrode, sodium ions in the sample undergo an ion exchange process with the sodium ions in the hydrated layer of the electrode. Changes in electrode potential occur as the ion exchange process takes place. These changes in electrode potential are referenced to the reference electrode. The "referenced potential" follows the Nernst equation and allows the calculation of sodium concentration in the sample: For more accurate measurement, the reference reagent containing sodium ions is introduced into the flow cell after the sample cycle, and the same ion exchange process takes place. The differential potential (voltage) between sample and reference reagent cycles is used for the calculation. Under ideal conditions, the electrode imparts a selectivity of 300:1 over potassium and is insensitive to hydrogen ions in solutions buffered from pH 6 to 10.

APPENDIX S3

APPENDIX S3-1: List of Food Items in Pure Study Software

S. No.	Items included in fruits	Items included	Items included	Other additional food items for calculating macro, micro-nutrients, vitamins and minerals	
	a	b	c	d	(a+b+c+d)
1	Banana	Ghia	Cornflakes	Chapati with Ghee	Egg gravy
2	Mango	Tori	Boiled Rice	Chapati dry	Chicken Gravy
3	Apple	Karela	Dhalia, all types	Bread	Chicken fried / roasted etc.
4	Water melon	Ladies finger (Bhindi)	Rusk	Toast	Mutton, pork, beef curries.
5	Peaches	Yam	Pasta	Bread rolls	Fish gravy
6	Pears	Capsicum	Biscuits, salted	Buns	Fish fried.
7	Orange	Tinda	Biscuits sweet, cream	Pulao / fried rice / zeera rice	Ham, salami, bacon etc.
8	Gauva	Saag	Chips, Khichre etc.	Plain Parantha	Bhel Puri
9	Papaya	Methi/Methey	Ice cream	Stuffed Parantha	Chats
10	Plum	Spinach	Cakes	Porridge	Patties
11	Grapes	Cabbage	Pastries	Puri	Pakoda
12	Musambi	Cauliflower	Custard	Bhature	Samosas
13	Pineapple	Brinjal	Kheer	Uppama	Mathies (NAMKEEN)
14	Pomegranete	Drumstick	Gulab Jamun	Poha	Namkeen, mixture etc.
15	Zizyphus	Colocasia	Jalebi	Noodles	Groundnuts
16		Fresh peas	Rasgulla	Macaroni	Cashew nuts
17		Kathal	Rasmalai	Pizza	Raitha with boondi
18		Beans	Sweet Mathi	Burger	Raitha with vegetables
19			Malpuda	Dosa	Namkeen lassi
20			Halwa	Idli	Whisky

S. No.	Items included in fruits	Items included	Items included	Other additional food items for calculating macro, micro-nutrients, vitamins and minerals	
	a	b	c	d	(a+b+c+d)
21			Carrrot Halwa	Khichidi	Beer
22			Ladoo	Rajma	Wine
23			Pinni	Saboot Moong	Cheese
24			Gujia	Whole gram curries-Black	Butter / Cream
25			Chocolates	Whole gram curries-White	Ghee
26			Candies	Dehusked dhal, all types	Added salt
27			Milk	Dhals with husk, all types	
28			Flavoured milk	Buttermilk curry	
29			TEA	Kofta curry	
30			Coffee	Green leafy vegetable curries	
31			Curd	Paneer gravy	
32			Sweet lassi	Mint / coriander chutney	
33			Fresh fruit juices	Tomato, tamrind, other chutneys	
34			Fruit juices packed	Veg/ Non veg soup	
35			Lemon water	Salad with raw vegetables	
36			Fanta, pepsi etc.	salad with sprouted grams	
37			Tomato Sauce	Papad Roasted	
38			Added sugar	Papad Fried	
39			Direct oral sugar intake	Pickle	
40			Jam	Boiled egg	
41			Jaggery	Bhurji	
42			Gur	Omlette	

APPENDIX S3-2: Micro-Nutrients, Vitamins and Minerals Estimation Using Pure Study Software

Example:

For example, if a participant has consumed two paranthas and a glass of milk in a day, their average portion size for parantha is two, and their frequency is one. For milk, portion size is one glass and frequency is one. When this data is entered into this software, the software estimates the nutrients based on the raw ingredients that are usually used for the preparation of the parantha. The software uses the information provided for raw ingredients for parantha as wheat flour, potatoes, oil, and spices. Based on the Indian dietary data provided by the National Institute of Nutrition, the software uses the weight of wheat flour, potato and oil in the two paranthas and a glass of milk. Further, as NIN provides data on the nutrients present per 100 g of food ingredients, the software uses the nutrients data given by NIN as per the quantity of ingredients used in preparation of the parantha. In two paranthas, it will estimate the result for proteins, fats and salt present in the ingredients of two paranthas and a glass of milk.

<table>
<tr><td colspan="3" rowspan="2">Breakfast</td><td colspan="2" rowspan="2">Estimated raw weight</td><td colspan="4">Calculated nutrient intake per 100 gram</td></tr>
<tr><td>Protein (g)</td><td>Carbohydrates (g)</td><td>Fat (g)</td><td>Energy (Kcal</td></tr>
<tr><td rowspan="2">Food Item</td><td rowspan="2">Stuffed parantha</td><td rowspan="2">Milk (I glass)</td><td rowspan="3">2 stuffed parantha</td><td>Wheat Flour =50g</td><td>5
2.5</td><td>33
16.5</td><td>0.7
0.35</td><td>158
79</td></tr>
<tr><td>Potato = 40g</td><td>0.6
0.24</td><td>9
3.6</td><td>0.4
0.16</td><td>42
16.8</td></tr>
<tr><td>Avg. Portion (quantity in which a food item is eaten at a time)</td><td>2</td><td>1</td><td>Oil=10ml</td><td></td><td>53
5.3</td><td>9
0.9</td><td>293
29.3</td></tr>
<tr><td rowspan="2">No. of times (no. of times a particular food item is eaten in a day)</td><td rowspan="2">1</td><td rowspan="2">1</td><td colspan="2">1 glass milk=150ml</td><td>20
30</td><td>12
18</td><td>34
51</td><td>434
651</td></tr>
<tr><td colspan="2">Total</td><td>25.6
32.74</td><td>107
43.4</td><td>44.1
52.41</td><td>927
776</td></tr>
</table>

APPENDIX S4

APPENDIX S4-1: Salt and Sugar Estimation

Salt

Sodium is estimated using methodology mentioned in annexure XII. Sodium estimated through the PURE study software was in mg/d. Sodium was convereted into salt g/d by using methodology adopted by previous studies97

Where,

Salt g/d= (Sodium mg/d × 2.54) ÷1000

Sugar

For sugar estimation, all the food items consumed by the participants, consisting of free and added sugars were clubbed together in an excel sheet (Annexure XI).

The grams of food in stanard size bowls, glass etc were estimated using data mentioned at 'Nutritionix'223.

Further, sugar in g/d for each of the food item based on the quantity as per the standard capacity of the bowls, glasses were estimated through 'Nutritionix database'223.

For each participant there were data on two dietary recalls colleceted on non-consequetive days and analysed separately.

The data of all the participants regarding the foods with addedd and free sugars were taken in a separate excel sheet. The data in the excel sheet was in the form of average quantity (quantity consumed at one meal, given in estimations of bowls, glasses etc) and frequency of consumption of the food items (number of times a particular food was consumed) on the day previous to the data collection.

In the excel sheet, the quantity and frequency of food items consumed by the participants were multiplied to get the total quantity (in estimations of no. of bowls, glasses etc.) of food consumed by partipants in one day.

These total quantity of all the food items consumed by the participants were multiplied by amount of sugar g/d (estimated as mentioned in point number 3.) to get the total sugar consumed by the participants

The sugar g/d were calculated for all the participants by this method. The sugar from two dietary recalls were calculated for each of the participant separately and then averaged to get the usual consumption of sugar in g/d.

Note: Standard nutrition database (Nutritionix) was used to provide the sugar information at the grocery stores and restaurants through an application programming interface (API).

Example:

A participant consumed one medium-sized banana twice in a day and one bowl of grapes in a day in the first recall. In the second recall, the participant consumed two medium-sized mangoes in a day (data are taken separately in excel sheet).

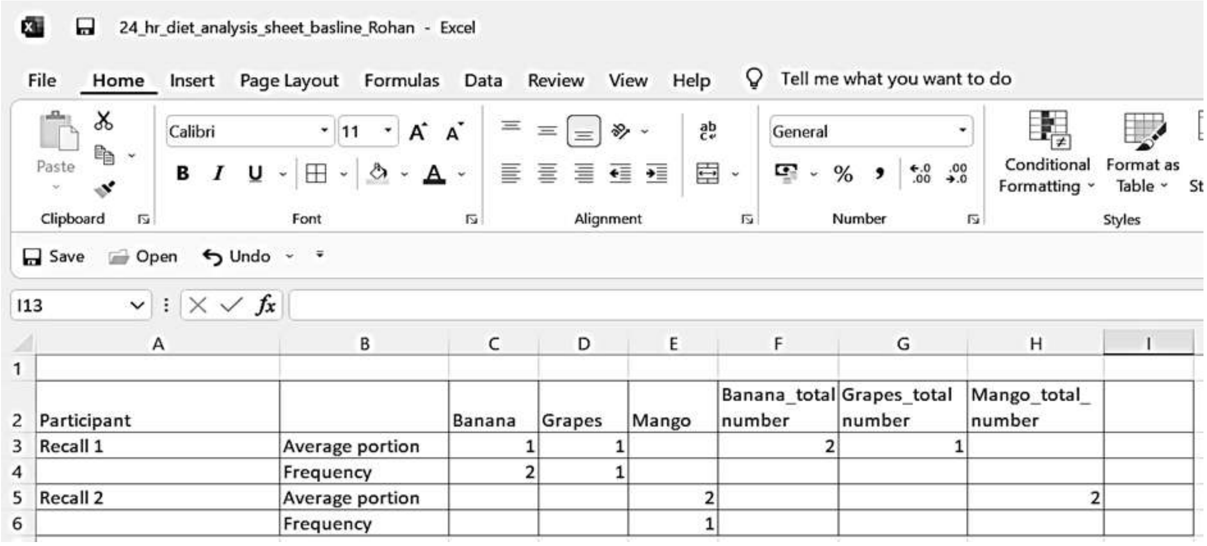

	A	B	C	D	E	F	G	H	I
1									
2	Participant		Banana	Grapes	Mango	Banana_total number	Grapes_total number	Mango_total_ number	
3	Recall 1	Average portion	1	1		2	1		
4		Frequency	2	1					
5	Recall 2	Average portion			2			2	
6		Frequency			1				

A total number of fruit portion was multiplied by 100 to get 200 g of banana and 100 g of grapes consumed by the participant in recall one and 200 g of mangos in recall two.

Recall one: 200+100+0 = 300g fruits/d

Recall two: 0+0+200= 200g fruits/d

Recall one and recall two averaged (300+200/2)= 250 g/d of fruits consumed by the participant.

This final mean value of 250 g/d is taken as the usual fruit consumed by the participant.

APPENDIX S5

APPENDIX S5-1: Participant Informed Assent Form

Project Title: Health Promotion Intervention Package for Prevention of Underlying Behavioral Risk Factors of Chronic Diseases: A Cluster Randomized Controlled Trial among Adolescents in School Settings

Principal Investigator: Ms. Sandeep Kaur

We are doing a research study to promote healthy behavioral practices among school going children, their parents & teachers to prevent risk factors of various chronic diseases. If you decide that you want to be part of this study, you will be asked to answer some health behavior related questions pertaining to dietary intake, physical activity, alcohol & tobacco use, etc .Your physical and anthropometric measurements will also be taken. We think this will take you around 30-45 minutes. Your blood pressure and blood glucose level will be measured.

No physical as well as psychological risks are involved in the present study.

Everyone who takes part in this study will benefit. A benefit means that something good happens to you. We think that the benefit for you will be that you will be get knowledge and learn the ways in which you can maintain your health and improve it by adapting to healthier behavioral practices and hence reducing the risk of getting these lifestyle diseases in future and in their maintenance, if already suffering from any.

When we are finished with this study we will write a report about what was learned. This report will not include your name or that you were in the study.

You do not have to be in this study if you do not want to be. You can stop participating at any time of the study. Your parents know about the study too.

If you decide you want to be in this study, please sign your name.

I, _________________________________, want to be in this research study.

___________________________________   _________

(Sign your name here)   (Date)

APPENDIX S5-2: Parental Informed Consent Form

Project title

Health Promotion Intervention Package for Prevention of Underlying Behavioral Risk Factors of Chronic Diseases: A Cluster Randomized Controlled Trial among Adolescents in School Settings

Introduction

Your child and you have been invited to join a research study entitled, 'Health Promotion Intervention Package for Prevention of Underlying Behavioral Risk Factors of Chronic Diseases: A Cluster Randomized Controlled Trial among Adolescents in School Settings' by Ms. Sandeep Kaur, PhD Scholar, School of Public Health, PGIMER, Chandigarh. Please take whatever time you need to discuss the study with your family and friends, or anyone else you wish to. The decision to let you and your child join, or not to join is up to you. In this research study, we are providing health promotion intervention to promote healthy behavioral practices among school going children, their parents & teachers to prevent the risk factors of various chronic diseases

What is involved in the study?

Your child and you will be asked to answer some health behavior related questions pertaining to dietary intake, physical activity, tobacco & alcohol use, etc. His/her and your physical and anthropometric measurements will also be taken. We think this will take him/her and you around 30-45 minutes each. Your child and yours blood pressure and blood glucose level will be measured. Your child can stop participating at any time. If your child stops he/she will not lose any benefits.

Risks

This study does not involve any physical and psychological risks.

Benefits of being a part of this study?

It is reasonable to expect the following benefits from this research: Benefit is that you and your child will get knowledge and learn the ways in which you can maintain your health and improve it by adapting to healthier behavioral practices and hence reducing the risk of getting these behavioral diseases in future and their maintenance, if already suffering from any.

Others may benefit in the future from the information we find in this study.

Confidentiality

Your child's and your name will not be used when data from this study are published but a code (a number) would be used purely for the purpose of monitoring the data collection process and will not be revealed beyond the study team. Every effort will be made to keep clinical records, research records, and other personal information confidential.

Your child rights as a research participant?

Participation in this study is voluntary. Your child has the right not to participate at all or to leave the study at any time. Deciding not to participate or choosing to leave the study will not result in any penalty or loss of benefits to which your child is entitled, and it will not harm his/her relationship with his/ her school authorities.

Permission for a Child to Participate in Research

As parent or legal guardian, I authorize ________________________________ (adolescent's name) to become a participant in the research study described in this form.

Adolescent's Date of Birth

Parent or Legal Guardian's Signature   Date

Consent to be a participant of the study

If you decide you want to be in this study, please sign your name.

I, ________________________________, want to be in this research study.

__________________________________   _________

(Sign your name here)   (Date)

APPENDIX S6: Number of Adolescent Participants for Each Indicator (%)

Indicator	Adolescents: n=462
Diet (24 hour recall)	453 (98%)
Anthropometric measurements	437 (95%)
24-hour urine samples	273 (59%)
Adolescents: out of 462 eligible, 9 were not recruited (2-not staying in UT Chandigarh for another year, 3 did not gave consent, 4 were enrolled in school but were not attending school)<br><br>Additional 16 adolescents did not consent to provide their anthropometric measures and 180 did not consent to provide 24-hour urine samples.	

APPENDIX S7

TABLE S1: Socio-demographic Characteristics of the Participating Adolescents (n=453)

Characteristics	n (%)
Sex	
Male	248 (55)
Female	205 (45)
Age (years)	
10-12	82 (18)
13-15	375 (81)
16-18	5 (1)
Religion	
Hindu	395 (87)
Muslim	17 (4)
Sikh	37 (8)
Others (Christian, Jain, Buddhist)	4 (1)
Caste	
General	299 (66)
Other backward class (OBC)	45 (10)
Scheduled caste (SC)	109 (24)
Scheduled tribe (ST)	0
Socio-economic status of household	
Lower	39 (8.6)
Upper Lower	277 (61)
Lower Middle	107 (24.2)
Upper Middle	29 (6)
Upper	1 (0.2)

APPENDIX S8

Table S2: Dietary Intake among Adolescents (n=453)

Socio-demographic Characteristics	Mean (SD) n=453							
	Salt (g/d)	P	Sugar (g/d)	P	Fruit (g/d)	P	Vegetable (g/d)	P
Overall	4.5 (1.9)		36 (33.7)		32.7 (79.5)		193.4 (172)	
Sex								
Male	4.6 (1.9)	0.2	33.6 (25)	0.01	25.3 (66.3)	0.03	168.6 (158.4)	0.001
Female	4.4 (1.8)		38.9 (41.6)		41.6 (92.3)		223.2 (183)	
Age(years)								
11-13	4.6 (1.9)	0.2	37(36)	0.2	32.5(79.6)	0.9	187(164)	0.2
14-16	4.3(1.6)		32(25)		33.2(79.3)		215(197)	
Socio-economic Status								
Lower	5.1 (2.8)	0.1	28 (24.7)	0.2	27.6 (62.3)	0.5	211.8 (194)	0.6
Upper Lower	4.4 (1.7)		35.1 (36)		31.2 (84.4)		199.5 (174.3)	
Lower Middle	4.7 (1.9)		37.1 (25.7)		34.1 (72.6)		184.4 (162.8)	
Upper Middle and Upper	4.8 (1.9)		46 (42)		50 (82.4)		152.6 (161.2)	

Recommended Dietary Allowance: Salt- 5 g/d (both sexes), sugar- 20 g/d for females, 25 g/d for males, fruits- 100 g/d (both sexes), vegetables. 300 g/d (both sexes)

Table S3: Urinary Excretory Salt Levels among the Adolescents

Socio-demographic characteristics	n(%) n=273				
	24-hour urine samples				
	Normal	High	Very High	Extremely high	P
Overall	16 (6)	39 (14)	84 (31)	134 (49)	
Sex					
Male	9 (6)	18 (12)	55 (36)	71 (46)	0.3
Female	67 (6)	21 (18)	29 (24)	63 (53)	
Age(years)					
11-13	31 (32)	51(53)	12 (12)	3(3)	0.2
14-16	8 (50)	7 (44)	1 (6)	0	
Socio-economic Status					
Lower	1 (5)	5 (23)	7 (32)	9 (41)	
Upper Lower	9 (5)	23 (13)	57 (33)	82 (48)	0.9
Lower Middle	5 (8)	9 (14)	15 (24)	34 (54)	
Upper Middle and Upper	0	2 (8)	6 (46)	6 (46)	

Excretory salt: Low=< 0.1 g/d, normal= < 2 g/d, high= ≥ 2 g/d to 4 g/d, very high= > 4 to 6 g/d and extremely high= > 6 g/d 28

Table S4: Anthropometric Measures among Adolescents

	Mean (SD): (n=437)									
Characteristics	BMI (Kg/m2)	P	WHR	P	MUAC (cm)	P	SSFT (mm)	P	TSFT (mm)	P
Overall	17.4 (4)		0.84 (0.06)		21.3 (3.3)		13.2 (7)		10.7 (6)	
Sex										
Male	17.2 (3)	0.2	0.85 (0.06)	0.001	21.1 (3.4)	0.2	12.3 (7)	0.001	9.7 (6)	<0.001
Female	17.7 (4)		0.83 (0.07)		21.5 (3.1)		14.4 (6)		12 (6)	
Age (years)										
10-12	17.2 (4)		0.83 (0.06)		21.3 (3.3)		13.4 (6)		10.5 (6)	
13-15	17.5 (3)	0.6	0.84 (0.06)	0.5	21.2 (3.3)	0.5	13 (7)	0.6	10.8 (6)	0.9
16-18	18.7 (3)		0.85 (0.06)		23 (4.8)		16 (3)		10.2 (1)	
Socio-economic Status										
Lower	17.5 (3.3)		0.84 (0.07)		21.6 (3.2)		12.3 (7)		11.5 (7)	
Upper Lower	17.3 (3.3)		0.84 (0.06)		21.1 (3.3)		12.8 (6)		10.3 (6)	
Lower Middle	17.5 (3.3)	0.1	0.84 (0.07)	0.7	21.4 (3.4)	0.5	14.4 (7)	0.1	11.2 (7)	0.3
Upper Middle and Upper	18.9 (5.9)		0.84 (0.06)		21.8 (3.3)		14.2 (7)		12 (6)	

APPENDIX S9

Table S5: Recommended Dietary Allowances of Macronutrients among Adolescents

Sex	Age (years)	Energy (Kcal /d)	Carbohydrates (g/d)	Protein (g/d)	Total fat (g/d)	Fibre (g/d)
Boys	10-12	2220	130	27	72	33
Girls	10-12	2060	121	27	67	30
Boys	13-15	2860	167	36	93	43
Girls	13-15	2400	140	35	78	36
Boys	16-18	3320	194	45	108	50
Girls	16-18	2500	146	37	81	38

Carbohydrates should be atleast 45% of the energy and fats 25% of the energy
1 Kcal=0.13g

Table S6: Recommended Dietary Allowances of Minerals among Adolescents

Sex	Age (years)	Calcium (mg/d)	Magnesium (mg/d)	Iron (mg/d)	Sodium (mg/d)	Potassium (mg/d)	Zinc (mg/d)	Iodine (µg/d)
Boys	10-12	650	199	12	2000	3500	7	70
Girls	10-12	650	207	16	2000	3500	7.1	70
Boys	13-15	800	287	15	2000	3500	11.9	100
Girls	13-15	800	282	17	2000	3500	10.7	100
Boys	16-18	850	367	18	2000	3500	14.7	100
Girls	16-18	850	317	18	2000	3500	11.8	100

Table S7: Recommended Dietary Allowances of Vitamins among Adolescents

Sex	Age (years)	Vit A (µg/d)	Thiamine (mg/d)	Riboflavin (mg/d)	Niacin (mg/d)	Vit B6 (mg/d)	Folate (µg/d)	Vit B 12 (µg/d)	Vit C (mg/d)
Boys	10-12	360	1.3	1.7	12	1.7	180	2	45
Girls	10-12	370	1.2	1.6	12	1.6	186	2	44
Boys	13-15	430	1.6	2.2	16	2.2	238	2	60
Girls	13-15	420	1.3	1.8	13	1.8	204	2	55
Boys	16-18	480	1.9	2.5	19	2.5	286	2	70
Girls	16-18	400	1.4	1.9	14	1.9	223	2	57

APPENDIX S10

Table S8: Percentage energy intake from carbohydrates and fats among Adolescents

	Mean (SD): (n=453)					
Characteristics	Energy (Kcal/d)	P	Carbohydrates (Kcal/d)	P	Total fat (Kcal/d)	P
Overall	1813 (594)		1176 (352)		423 (234)	
Sex						
Male	1838 (570)	0.3	1188 (332)	0.4	423 (234)	0.7
Female	1782 (622)		1160 (376)		414 (234)	
Age (years)						
10-12	1738 (516)		1112 (288)		414 (207)	
13-15	1829 (602)	0.5	1188 (360)	0.2	423 (234)	0.9
16-18	1852 (1109)		1192 (600)		450 (414)	

Cut-offs for all macronutrients, minerals and vitamins intake depicted in the figure were adopted from Recommended Dietary Allowance (RDA) for the Indian populations from the Indian Council of Medical Research-National Institute of Nutrition (ICMR-NIN), Hyderabad (2020)22
