# Supplementary material for: Nutritional assessment of adolescents: A cross-sectional study from public schools of North India
Source: PLoS One. 2025 Jan 14;20(1):e0316435. doi: 10.1371/journal.pone.0316435 (PMC11731870; doi:10.1371/journal.pone.0316435)
Supplement: Attachment — Submitted filename: nutrtional assessments -PONE-D-23-18349.pdf [file pone.0316435.s004.pdf]

PLOS ONE

Nutritional Assessment of Adolescents: a Cross-sectional Study from Public Schools of North India

--Manuscript Draft--

**Manuscript Number:**	PONE-D-23-18349
**Article Type:**	Research Article
**Full Title:**	Nutritional Assessment of Adolescents: a Cross-sectional Study from Public Schools of North India
**Short Title:**	Nutrition status of public-school going adolescents from North India
**Corresponding Author:**	Sandeep Kaur, Ph.D.<br>Imperial College London - Hammersmith Campus<br>London, UNITED KINGDOM
**Keywords:**	Nutritional status, chronic diseases, diet, anthropometric, adolescents, urban, socio-economic status
**Abstract:**	Background: Technological advancements and globalisation has led to a shift in dietary behaviours and nutrition patterns. In recent years there has also been an alarming increase in the prevalence of chronic diseases in LMICs, including India. Adolescents are more susceptible to the negative impact of changing dietary practices. Furthermore, the nutritional status in the early years of adolescence becomes the basis of their overall health during adulthood. The present study, was designed to assess the nutritional status of adolescents in public schools in the North Indian city of Chandigarh, India.<br>Methodology: The present study was based on a cross-sectional survey undertaken as part of a larger cluster randomised control trial (CRCT). A two-stage random sampling technique was used to select 12 schools and a eighth-grade class. A total of 453 adolescents in the age group of 10-16 years were recruited. Nutritional status was measured by assessing the selected adolescents' dietary behavior, anthropometric measures and 24-hour urinary excretory salt-levels. Dietary pattern was recorded using two 24-hour dietary recalls on non-consecutive days. Anthropometric measures were recorded using standardised tools and methods. The 24-hour urinary excretory salt levels were assessed through standardized laboratory methods.<br>Results: The mean age of adolescents was 13.06 years, and 55% were boys. 32 % of adolescents had high salt, and 55% had high sugar intake. Whereas, 90% of adolescents had low fruit and 83% had low vegetable intake. Adolescents were deficient in most macro and micro-nutrients, such as energy, fats, fibre, iron, zinc, iodine, riboflavin, and vitamins B-6 and B-12. More proportions of boys (10%) were thin compared to girls (2%). Higher proportions of girls had abdominal obesity (36%). In contrast, high proportions of boys were severely acute malnourished (23%). Almost all adolescents had high urinary excretory salt levels.<br>Conclusion: Most adolescents had dietary risk factors, as high intake of salt and sugar and low consumption of fruits and vegetables. Most of adolescents were deficient in various macro and micro-nutrients, with the co-existence of thinness and obesity. Schools must regularly assess adolescents' nutritional status to address this double whammy of nutrition. Additionally, health-promoting intervention needs to be developed in school settings to promote healthy dietary practices.
**Order of Authors:**	Sandeep Kaur, Ph.D.
	Rajesh Kumar, MD
	Manmeet Kaur, Ph.D.
**Additional Information:**	
**Question**	**Response**
**Financial Disclosure**<br><br>Enter a financial disclosure statement that	Partial funding was awarded to MK with<br>project ID 5562 for undertaking laboratory tests of 24-hour urine samples from The George Institute for Global Health.

Powered by Editorial Manager® and ProduXion Manager® from Aries Systems Corporation

describes the sources of funding for the work included in this submission. Review the submission guidelines for detailed requirements. View published research articles from *PLOS ONE* for specific examples.<br><br>This statement is required for submission and **will appear in the published article** if the submission is accepted. Please make sure it is accurate.<br><br>**Unfunded studies**<br>Enter: *The author(s) received no specific funding for this work.*<br><br>**Funded studies**<br>Enter a statement with the following details:<br>• Initials of the authors who received each award<br>• Grant numbers awarded to each author<br>• The full name of each funder<br>• URL of each funder website<br>• Did the sponsors or funders play any role in the study design, data collection and analysis, decision to publish, or preparation of the manuscript?<br>• **NO** - Include this sentence at the end of your statement: *The funders had no role in study design, data collection and analysis, decision to publish, or preparation of the manuscript.*<br>• **YES** - Specify the role(s) played.<br><br>* typeset	The funders had no role in study design, data collection and analysis, decision to publish, or preparation of the manuscript.
**Competing Interests**<br><br>Use the instructions below to enter a competing interest statement for this submission. On behalf of all authors, disclose any competing interests that could be perceived to bias this work—acknowledging all financial support and any other relevant financial or non-financial competing interests.<br><br>This statement is required for submission and **will appear in the published article** if the submission is accepted. Please make sure it is accurate and that any funding	The authors have declared that no competing interests exist.

sources listed in your Funding Information later in the submission form are also declared in your Financial Disclosure statement.<br><br>View published research articles from *PLOS ONE* for specific examples.<br><br>**NO authors have competing interests**<br><br>Enter: *The authors have declared that no competing interests exist.*<br><br>**Authors with competing interests**<br><br>Enter competing interest details beginning with this statement:<br><br>*I have read the journal's policy and the authors of this manuscript have the following competing interests: [insert competing interests here]*<br><br>* typeset	
**Ethics Statement**<br><br>Enter an ethics statement for this submission. This statement is required if the study involved:<br><br>• Human participants<br>• Human specimens or tissue<br>• Vertebrate animals or cephalopods<br>• Vertebrate embryos or tissues<br>• Field research<br><br>Write "N/A" if the submission does not require an ethics statement.<br><br>General guidance is provided below. Consult the submission guidelines for detailed instructions. **Make sure that all information entered here is included in the Methods section of the manuscript.**	Ethical permission has been obtained from the Post-Graduate Institute of Medical Education and Research, Chandigarh (INT/IEC/2018/000082; Date: 22/ 01/2019).<br><br>Written assent of adolescents and written consent from their parents were obtained after informing them about the study purpose, data to be collected, estimated time for data collection, the confidentiality of the data, and risks involved before their enrolment in the study. A sample form of the assent and consent form used in the study has been provided in the supplement as Appendix S6.

Powered by Editorial Manager® and ProduXion Manager® from Aries Systems Corporation

**Format for specific study types**<br><br>**Human Subject Research (involving human participants and/or tissue)**<br>• Give the name of the institutional review board or ethics committee that approved the study<br>• Include the approval number and/or a statement indicating approval of this research<br>• Indicate the form of consent obtained (written/oral) or the reason that consent was not obtained (e.g. the data were analyzed anonymously)<br><br>**Animal Research (involving vertebrate animals, embryos or tissues)**<br>• Provide the name of the Institutional Animal Care and Use Committee (IACUC) or other relevant ethics board that reviewed the study protocol, and indicate whether they approved this research or granted a formal waiver of ethical approval<br>• Include an approval number if one was obtained<br>• If the study involved *non-human primates*, add *additional details* about animal welfare and steps taken to ameliorate suffering<br>• If anesthesia, euthanasia, or any kind of animal sacrifice is part of the study, include briefly which substances and/or methods were applied<br><br>**Field Research**<br><br>Include the following details if this study involves the collection of plant, animal, or other materials from a natural setting:<br>• Field permit number<br>• Name of the institution or relevant body that granted permission	
**Data Availability**<br><br>Authors are required to make all data underlying the findings described fully available, without restriction, and from the time of publication. PLOS allows rare exceptions to address legal and ethical concerns. See the PLOS Data Policy and FAQ for detailed information.	Yes - all data are fully available without restriction

A Data Availability Statement describing where the data can be found is required at submission. Your answers to this question constitute the Data Availability Statement and **will be published in the article**, if accepted.<br><br>**Important:** Stating 'data available on request from the author' is not sufficient. If your data are only available upon request, select 'No' for the first question and explain your exceptional situation in the text box.<br><br>Do the authors confirm that all data underlying the findings described in their manuscript are fully available without restriction?	
**Describe where the data may be found in full sentences. If you are copying our sample text, replace any instances of XXX with the appropriate details.**<br><br>• If the data are **held or will be held in a public repository**, include URLs, accession numbers or DOIs. If this information will only be available after acceptance, indicate this by ticking the box below. For example: *All XXX files are available from the XXX database (accession number(s) XXX, XXX.*).<br>• If the data are all contained **within the manuscript and/or Supporting Information files**, enter the following: *All relevant data are within the manuscript and its Supporting Information files.*<br>• If neither of these applies but you are able to provide **details of access elsewhere**, with or without limitations, please do so. For example:<br><br>*Data cannot be shared publicly because of [XXX]. Data are available from the XXX Institutional Data Access / Ethics Committee (contact via XXX) for researchers who meet the criteria for access to confidential data.*<br><br>*The data underlying the results presented in the study are available from (include the name of the third party*	No later than three years after collecting the 1-year end-line assessment, we will deliver a wholly deidentified data set to an appropriate data archive for sharing purposes.

and contact information or URL).<br>• This text is appropriate if the data are owned by a third party and authors do not have permission to share the data.<br><br>* typeset	
Additional data availability information:	Tick here if the URLs/accession numbers/DOIs will be available only after acceptance of the manuscript for publication so that we can ensure their inclusion before publication.

Manuscript Click here to access/download;Manuscript;Manuscript.docx

Nutritional Assessment of Adolescents: a Cross-sectional Study from Public Schools of North India

Sandeep Kaur[1,2*], Rajesh Kumar[1], Manmeet Kaur[1]

[1] Department of Community Medicine and School of Public Health, Post-Graduate Institute of Medical Education and Research, Chandigarh, 160012, India

[2] Department of Surgery and Cancer, Faculty of Medicine, Imperial College London, Hammersmith Campus, London W12 0NN

***Corresponding Author**

Email: sandeep.kaur@imperial.ac.uk

Authors contribution

SK conceptualised the study, carried out the data collection and curation and its formal analysis and prepared and wrote the original draft of the manuscript. RK and MK provided their supervision, contributed in conceptualizing the study, validated the data and results, and provided the resources required to carry out this study. All authors were involved in reviewing and editing the manuscript and gave their final approval for the version to be published.

Conflict of Interest

The authors declare no conflict of interest.

ABSTRACT

Title: Nutritional Assessment of Adolescents: a Cross-sectional Study from Public Schools of North India

Background: Technological advancements and globalisation has led to a shift in dietary behaviours and nutrition patterns. In recent years there has also been an alarming increase in the prevalence of chronic diseases in LMICs, including India. Adolescents are more susceptible to the negative impact of changing dietary practices. Furthermore, the nutritional status in the early years of adolescence becomes the basis of their overall health during adulthood. The present study, was designed to assess the nutritional status of adolescents in public schools in the North Indian city of Chandigarh, India.

Methodology: The present study was based on a cross-sectional survey undertaken as part of a larger cluster randomised control trial (CRCT). A two-stage random sampling technique was used to select 12 schools and a eighth-grade class. A total of 453 adolescents in the age group of 10-16 years were recruited. Nutritional status was measured by assessing the selected adolescents' dietary behavior, anthropometric measures and 24-hour urinary excretory salt-levels. Dietary pattern was recorded using two 24-hour dietary recalls on non-consecutive days. Anthropometric measures were recorded using standardised tools and methods. The 24-hour urinary excretory salt levels were assessed through standardized laboratory methods.

Results: The mean age of adolescents was 13.06 years, and 55% were boys. 32 % of adolescents had high salt, and 55% had high sugar intake. Whereas, 90% of adolescents had low fruit and 83% had low vegetable intake. Adolescents were deficient in most macro and micro-nutrients, such as energy, fats, fibre, iron, zinc, iodine, riboflavin, and vitamins B-6 and B-12. More proportions of boys (10%) were thin compared to girls (2%). Higher proportions of girls had abdominal obesity (36%). In contrast, high proportions of boys were severely acute malnourished (23%). Almost all adolescents had high urinary excretory salt levels.

Conclusion: Most adolescents had dietary risk factors, as high intake of salt and sugar and low consumption of fruits and vegetables. Most of adolescents were deficient in various macro and micro-nutrients, with the co-existence of thinness and obesity. Schools must regularly assess adolescents' nutritional status to address this double whammy of nutrition. Additionally, health-promoting intervention needs to be developed in school settings to promote healthy dietary practices.

Keywords: Nutritional status, chronic diseases, diet, anthropometric, adolescents, urban, socio-economic status.

Nutritional Assessment of Adolescents: a Cross-sectional Study from Public Schools of Chandigarh, India

INTRODUCTION

Technological advancements, easy availability, and tempting advertisements of processed foods allure young people, especially adolescents, to consume energy-dense foods with low nutritional value, finally leading to a buildup of unhealthy dietary behaviours in adulthood.[1]

Healthy dietary behaviours have been as low as 40% worldwide, with scenarios marginally better in high-income countries (HIC) compared to low-and-middle-income countries (LMICs).[2] Among LMICs, India's situation is gruesome, with only 10.6% of Indians following healthy dietary behaviours.[3] India is in the midst of a global transition, wherein it faces the double burden of both under-nutrition and over-nutrition.[4] A recent NFHS-5 survey (15-49 years) revealed that 23% of women and 20% of men were underweight.[5] In comparison, 21% of women and 19% of men were overweight or obese.[5] These findings signify the co-existence of underweight and overweight among the Indian population.[5]

This increased prevalence of malnutrition has also been associated with setting the epidemiological transition in motion, i.e., the proportional increase in the prevalence of chronic diseases and their associated morbidity and mortality.[4,6]As, in the same NFHS-5 survey, 14% of women and 16% of men had high blood glucose levels and 21% of women and 24% of men had high blood pressure levels.[5]

The behaviour of unhealthy dietary practices leading to malnourishment emerges during early adolescence.[1] When these behaviours are not addressed timely, the early year of malnourishment becomes one of the most significant risk factors in the occurrence of chronic diseases in later life.[6,7] Adolescence is a transition phase of human life, both physically and socially.[7] At this age, the quality of nutrition is one of the important factors leading to the holistic growth of adolescents into healthy adults.[8]

It is the need of the hour to regularly assess nutrition behaviour during early adolescence to address the issue of malnutrition and keep a check on one of the risk factors of chronic diseases.[1,6] Owing to expensive, lifelong treatments and loss of productive life years due to the increasing premature deaths and disabilities related to various chronic diseases in India[9], it is recommended to focus more on promotion and prevention than curative measures. Therefore, as a part of a larger comprehensive behaviour-change intervention, the present cross-sectional study aims to assess the nutrition status and anthropometric measures of early adolescents studying in the public schools of North India.

METHODS

Study design

The present analysis is based on a cross-sectional survey carried out as part of a broader cluster randomized control trial to assess the impact of a school-based health promotion intervention package in reducing the behavioural risk factors associated with chronic diseases.[10] The survey was conducted among adolescents studying in the public schools of the North Indian city of Chandigarh, India from May 2018 to September 2019.

Study setting

The study was conducted in public schools as the Director of Public Instruction did not allow enrolment of private schools in the study. Data collected from the Department of School Education Chandigarh revealed that 106 public schools had eighth-grade classes, and on average, 10.790 adolescents study in 8th grade in these schools. Most schools had four to five sections in 8th-grade class. Each section of the class had about 30 adolescents.

Participants

Based on the knowledge that behaviours leading to malnourishment emerge during early adolescence and analysis of the school curriculum, consenting adolescents between the ages of 10-16 from eight-grade were included in the present study.[7] An additional inclusion criterion was the adolescents not intending to leave the city for another year from the recruitment date.

Sample size and sampling

Details on the sample size calculation are mentioned in the protocol paper of the broader CRCT study.[10] Broadly, the required number of clusters for 80% power for each primary outcome was calculated separately using expected means or proportions for the control arm, intervention effects, and coefficient of variation values based on data for adolescents from recent studies.[11-15] Out of all risk factors, the maximum ‘c’ value (n=12) was considered the sample size of the number of clusters for the study.[10] From the calculated sample of 12 clusters (schools), and cluster size of 30 adolescents, a final sample size of 360 adolescents was estimated.

Outcome variables

Nutritional status, anthropometric measurements and 24-hour excretory salt levels were the primary outcome variables. Nutritional status was assessed by estimating the dietary intake of salt, sugar, fruits and vegetables in g/d, along with macronutrients (energy, carbohydrates, proteins, fats and fibre), minerals (calcium, magnesium, iron, sodium, potassium, zinc and iodine) and vitamins (vitamin A, thiamine, riboflavin, niacin, vitamin- B6, folate, vitamin B-12 and vitamin C) intake. In addition to nutritional assessment, salt was also assessed through 24-hour urine samples in the form of excretory-urinary salt levels.

Anthropometric measurements estimated the prevalence of severe thiness, thiness, overweight, obesity, abdominal obesity and severely acute malnourishment among the selected adolescents.

Data collection tools

Nutritional status was measured through the administration of a 24-hour dietary recall questionnaire. This questionnaire was administered twice on non-consecutive days.[16] Data collected through the 24-hour dietary recall was analysed using the PURE study software. This software uses the National Institute of Nutrition (NIN) dietary guidelines for Indians to calculate the quantity of various macro and micronutrients from the quantity of various food items consumed by a person in a day.[17,18] Socio-economic status (SES) was assessed using the 'Kuppuswamy 2018 scale'.[19] This scale uses information about the education and occupation of the head of the household and the household's total monthly income to score and rank participants into five categories of socio-economic status, i.e., lower, upper-lower, lower-middle, upper-middle, and upper. [19]

Height was measured with UNICEF's standardised anthropometer to the nearest 0.1 cm. Weight was measured with a portable electronic weighing scale to the nearest 0.1 kg. Hip circumference (HC), waist circumference (WC), and Mid Upper Arm Circumference (MUAC) was measured to the nearest 0.1 cm by using fibreglass measuring tape. Triceps Skin-fold Thickness (TSFT) and Sub Scapular Skin-fold Thickness (SSFT) were measured to the nearest 0.1 mm using Lange's skin-fold calliper.

For 24-hour urine samples, adolescents were advised to write the time of their voids and whether they have collected each void in the collection container or not for 24 hours using a structured proforma (Appendix S1).

Data collection methods

SK recorded the dietary and anthropometric data. A team of three members, including SK, collected 24-hour urine samples in the same time period from May 2018 to September 2019. Standard operating procedures were developed to maintain uniformity and standardization in sample collection. The self-administered 24-hour dietary recalls were recorded for all adolescents.[16] SK was present to explain each question to adolescents before they recorded their responses. The first 24-hour dietary recall collection was during the first interaction with all the adolescents in the schools. The second 24-hour dietary recall collection was a week later. Anthropometric measures were recorded in a separate room after recording dietary data.

All anthropometric instruments were regularly calibrated as per standard requirements. The portable digital weighing scale was calibrated after every 20th reading. ‘Lange’s’ skinfold calliper was checked for needle at ‘zero’ before every reading and was calibrated using a calibration block after every 20th reading. Fibreglass tape was used to measure hip, waist and mid-upper-arm-circumference as it is resilient to wear and tear and does not expand in warm weather conditions like an ordinary measuring tape[20]. SK was trained to record anthropometric measurements during the Comprehensive National Nutrition Survey (CNNS)-2018 training. A senior faculty member working in health promotion did the quality control of the data collection process. In addition, a Doctoral Committee comprising of seven faculty members of the institute, working independent from the sponsor and competing interests, were explicitly constituted for data monitoring and progress evaluation of this project.

The 24-hour urine samples were collected during house visits. During Saturdays' home visits, participants received instructions regarding the process of 24-hour urine collection on Sundays. Urine collection containers were provided to them. They were instructed to discard the first void of the morning and then start collecting the subsequent voids for the next 24 hours (Appendix S2). The urine samples were collected on Monday mornings and analysed in Beckman Coulter AU 5800 analyzer (2020) using standardized laboratory methods to assess urinary salt levels on the same day (Appendix S2).

Data management and analysis

The PURE study computer software was used to analyse the diet data and estimate the intake of macro and micro-nutrients (i.e., protein, carbohydrates, fat, vitamins, and minerals).[21] This tool has been developed explicitly for assessing the intake of Indian foods. It has been previously validated in Indian settings; from this software, 72 different macro and micro-nutrients can be estimated (Appendix S3). The widely used and accepted guidelines for Recommended Dietary Allowance (RDA) for the Indian populations from the Indian Council of Medical Research-

National Institute of Nutrition (ICMR-NIN), Hyderabad (2020), were used to compare the dietary nutritional status of the adolescents estimated in the present study.[22] The twenty dietary nutrients considered essential for young adolescents' proper growth and health, as laid down by ICMR-NIN, were used to assess the dietary nutrition for the present study.[22] The method of calculating salt, sugar, fruits and vegetables in g/d from 24-hour dietary recall is presented in the supplement (Appendix S4)

Adolescents' body mass index (BMI) was used to categorise adolescents into severe thin, thin, normal, overweight and obese categories based on WHO growth reference percentiles that were age and sex-specific.[23] For waist-hip ratio (WHR), WHO cut-offs were used for all adolescents.[24] Age and sex-specific percentiles were used to categorise mid-upper arm circumference (MUAC), triceps skinfold thickness (TSFT) and sub-scapular skinfold thickness (SSFT).[25]

The complete quantitative data analysis was performed using the Statistical Package for Social Sciences (SPSS) version 21 and Microsoft Excel 2007.[26]. Descriptive statistical analysis includes the estimation of the sample mean (along with standard deviation) or proportions according to the variable types. As, the sample size was sufficient to test these hypotheses, unpaired-t or chi-square tests were used to explore potential differences between groups (e.g., age, gender and socio-economic status). ANOVA was used to test the statistical significance of continuous variables in bivariate analysis. P-value <0.05 was considered to be statistically significant.

The cut-offs to categorize all the adolescents into low, normal, high, very high and extremely high categories were used from literature to assess urinary salt excretion from 24-hour urine samples.[27]

The authors have access to the participants' identifiable information, with access to this data limited to them. However, wholly de-identified data will be shared at an appropriate data archive for sharing purposes after three years of the end-line assessment data collection as this study is part of a larger cluster Randomized Controlled Trial.

Ethical considerations

Ethical permission was obtained from the Institutional Ethics Committee of the Post-Graduate Institute of Medical Education and Research, Chandigarh (INT/IEC/2018/ 000082; Date. 22/ 01/2019) (Appendix S5). Permission was granted for the study in the schools by the Department of Education, Chandigarh, with letter no 1296-DSE-UT-S5-11(65)11-II. As the present study was part of a cluster randomised controlled trial, the study was registered in CTRI with no. CTRI/2019/09/021452.

Written assent of adolescents and written consent from their parents were obtained after informing them about the study purpose, data to be collected, estimated time for data collection, the confidentiality of the data, and risks involved before their enrolment in the study (Appendix S6).

RESULTS

Out of the 462 eligible adolescents from twelve randomly selected public schools, 453 consented to participate in this cross-sectional survey (Appendix S7). Out of selected adolescents, 55% were boys, 81% were 13-15 years old (81%), 87% belonged to the Hindu religion, 66% were

from the general category, and 61% belonged to upper-lower socio-economic status category (Table 1).

Table 1: Socio-demographic characteristics of the study adolescents (n=453)

The consumption of salt, sugar, fruit and vegetables was also estimated separately (Appendix S8: Table S1). The mean consumption of salt was 4.5 g/d which was within the RDA. The mean intake of sugar was 36 g/d. It was lower (34 g/d) among boys than girls (39 g/d).

The mean vegetable intake (193 g/d) was lower than the RDA of 300 g/d. The mean vegetable intake among boys was lower (169 g/d) than the girls (223 g/d). The average intake of fruits was 32.7 g/d, which is less than the RDA of 100 g/d (Appendix S8: Table S1).

Figure 1. Adequacy of dietary intakes of salt, sugar, fruits and vegetables among adolescents (n=453)

Thirty-two per cent and 55% of adolescents consumed higher salt and sugar than the RDA. At the same time, the consumption of fruits and vegetables was poor, with 90% and 83% of adolescents consuming low levels. Therefore, it was necessary to implement health promotion interventions to change these dietary behaviours (Figure 1).

Ninety-four percent of adolescents had high to extremely high excretory salt levels based on the 24-hour urine samples. There was no difference in its distribution in sex, age and socio-economic category (Appendix S8: Table S2).

Table 2: Macro-Nutrient Intake among Adolescents

The RDA for adolescents specific to their age and sex were adopted from Recommended Dietary Allowance (RDA) for the Indian populations from the Indian Council of Medical Research-National Institute of Nutrition (ICMR-NIN), Hyderabad (2020).[22] The average daily energy intake was less than the RDA in both sexes. The carbohydrate consumption was above the dietary guidelines. Likewise, protein intake was adequate in both boys and girls. There was gender specific sta[illegible]ically significant difference in protein intake, with boys consuming more. The mean fat intake among adolescents was 47 g/d and it was within RDA for both boys and girls. The fibre intake was lower than the RDA for both boys and girls (Table 2).

Table 3: Mineral Intake among Adolescents

Adolescents' overall mean calcium, magnesium and iron intake was less than the RDA. Mean sodium intake was within the RDA of 2000 mg/d. Potassium intake was less than the RDA of ≥3500 mg/d with a mean of 1954 mg/d. Iodine intake assessed from the consumed food had a mean of 67 µg/d, less than half of the RDA of ≥150 µg/d. Mean zinc intake was well within the RDA, and mean iodine intake was near the RDA (Table 3).

Table 4: Vitamin Intake among Adolescents

Vitamin A consumption was lower than the RDA for both sexes. Thiamine and niacin intake were well within the RDA, but riboflavin and vitamin B-6 were slightly below the RDA. Folate consumption was within the RDA for both sexes. However, boys consumed significantly more

folate than girls. Consumption of vitamin B-12 was insufficient compared to the RDA. The average vitamin C intake was not as per the RDA among adolescents. It was found that girls consumed more vitamin C (Table 4).

Figure 2: Proportions of adolescents with adequate intake of nutrients (n=453)

Most adolescents were deficient in the major macro and micronutrients (Figure 2). Carbohydrate was the only macronutrient that was adequate in almost all adolescents. Iodine, fibre, potassium, vitamin B-12 and calcium were the highly deficient nutrients in most adolescents (Figure 2).

Anthropometric measures

The mean BMI was 17 kg/m^2, which was within the normal range compared to the WHO reference curves of adolescents.[23] The waist-hip ratio among boys was 0.85, which was less than the cut-off of 0.9 for males.[24] Similarly, it was 0.83 for girls, which was slightly less than the cut-off of 0.85 for females signifying normal abdominal obesity among boys and a girls (Appendix S8: Table S3).[24]

Average Mid-Upper Arm Circumference (MUAC) was 21.3 cm. Boys had nearly 2 cm less than the normal range of 23 cm, and girls had 0.5 cm less than the normal range of 22 cm.[28] It signifies that both the sexes were under-nourished, boys being thinner than the girls. MUAC also has a significant difference among the age groups. The young adolescents had lesser MUAC compared to the older.

Skin-fold thicknesses are a robust method for the assessment of subcutaneous fat. The average TSFT and SSFT were 13.2 mm and 10.7 mm, respectively. These values were in the normal range compared to the reference curves of Indian adolescents (5-17 years).[25] There was a significant difference among both sexes for the skin folds, with girls having a higher mean for both the TSFT (14.4 mm) and SSFT (12 mm).

Figure 3: Malnourishment assessed through anthropometric measures

Among adolescents, the prevalence of thinness and obesity co-existed. More proportions of boys were thin. Whereas, more proportions of girls were in overweight and obese BMI category. On similar lines, more girls were found to have abdominal obesity. The similar proportion of adolescent boys and girls had high subcutaneous fats assessed through triceps and sub-scapular skinfold thicknesses. A finding in line with another anthropometric measure, MUAC, where higher proportions of boys were found to have severe acute malnutrition (Figure 3).

DISCUSSION

The nutritional assessment revealed that the risk factors associated with chronic diseases were high in the study population. Among adolescents studying in eighth grade, 32% and 55% consumed high salt and sugar than the RDA, respectively, and the consumption of fruits (90%) and vegetables (83%) was inadequate in most of them. Further, it was revealed that most of the adolescents were deficient in majority of the macro and micronutrients. There was co-existence of over and undernutrition among the adolescents with more proportions of girls being overweight, and having high BMI and abdominal obesity compared to their male counterparts.

The mean value of 24-hour urinary excretory salt levels through 24-hour urine samples, which is considered the gold standard, was 7 g/d.

Adolescents had energy intake below the RDA. This finding was consistent with other global and Indian studies among adolescents.[29,30] Boys had a significantly higher intake of proteins than girls, and this finding was consistent with another study among adolescents from other LMICs.[31] Fat intake among adolescents in this study was within the RDA. This finding was in line with another study from India. For girls, the fat intake was slightly higher than the RDA, a result in line with other global studies.[31,32]

This study indicated that intake of iron (15 mg/d), potassium (1954 mg/d), vitamin A (2497 µg/d), and iodine (67 µg/d) was below the RDA. National Nutrition Monitoring Bureau (NNMB) have also documented that micronutrient intakes were inadequate among Indian adolescents.[33] These findings are comparable to studies from HICs.[33,34] Generally, insufficient micronutrients, such as iron, have been attributed to a low intake of animal-source foods and potassium and vitamin A to the low consumption of fruits and vegetables. Most of the adolescents from the present study belonged to the low SES groups, where affordability could have been a significant reason for poor micronutrient intakes.[34]

The previous studies among adolescents reveal that the mean iodine intake increased by 20% when data was adjusted for the intake of iodised salt.[32] The low level of Iodine intake estimated in the present study can be attributed to the non-inclusion of iodised salt intake in the dietary data analysis. Future studies may include analysis through more accurate measures such as urinary iodine.

The 'BMI for age' is recommended by WHO as the best indicator to assess under-nutrition (thinness) or overweight in adolescents. The present study has adopted the standard methods for determining the nutritional status of adolescents as recommended by the WHO. Various studies across India have adopted the same method for evaluating the nutritional status of adolescents.[35]

Underweight (24%) and overweight (10%) co-existed in the present study. Another study from LMIC has reported higher under-nutrition prevalence (45%).[36] But, the finding was consistent with Indian studies.[35,37] The prevalence of overweight or obesity in the present study was 12%. Indian studies have reported similar results.[35,37] The increased prevalence of overweight may be attributed to the increasing accessibility of processed foods and motorised transportation modes. The prevalence of overweight and obesity was higher among girls (12%) than boys (9%). This finding is similar to the studies conducted across the country.[38,39] In contrast, few studies also show a higher prevalence of overweight and obesity among boys when compared to girls.[35,38,40]

Most of the anthropometric indices had higher values among boys. This is supported by the fact that boys usually have a larger body build at this age (10 to 15 years), grow to a larger structure and continue to increase more in height than the girls even after adolescence.[40,41] Several authors also observed a similar trend from their studies in other states of India and abroad.[42] The difference in the nutritional status assessed through different studies may be due to different cultural environments, the difference in tools used for data collection, and their methodology and analysis. Other classification criteria may also lead to differences in the results. Various classifications such as CDC guidelines 2000, International Obesity Task Force and WHO BMI

reference charts etc., are available for estimating nutritional status. Additionally, socio-economic and socio-demographic factors and their associated risk factors may also lead to variation in the results.

The boys' mean WHR was more than that of girls. The mean WHR of the boys and girls were below the WHR ratio cut-offs of 0.85 and 0.9 respectively, signifying normal abdominal obesity. This finding was in line with previous studies.[43]

The average TSFT and SSFT were 13.2 mm and 10.7 mm, respectively. The reference curves of Indian adolescents aged (5-17 years) signify that it is in the normal range of subcutaneous fat.[25] Findings were consistent with a study among adolescents in the Netherlands.[44] Average values for both TSFT and SSFT were higher among girls. These findings were consistent with other studies carried out among adolescents of a similar age group in India, Vietnam and Indonesia.[45-47] The findings of 24-hour urinary excretory salt levels from the present study is compared with another study carried out in China among the adolescents.[48]

The food frequency questionnaire (FFQ) and the 24-hour dietary recall were the commonly used tools in most studies assessing the participants' dietary behaviours as these are quick and inexpensive to administer and have high respondent compliance. From the previous studies, it is evident that FFQ has a higher recall bias compared to the 24-hour dietary recall. The 24-hour dietary recalls have been known to estimate population-level consumption but not accurate individual-level consumption patterns.[16] To overcome this limitation, two 24-hour dietary recalls were recorded on the non-consecutive days for the present study. The two recalls were collected with a time gap of one week to one month. The multiple-pass method was used to collect the dietary behaviours as precise as possible. Standard household measures (e.g. spoons, bowls, glasses and plates) were used to generate actual quantities of ingredients. Another limitation of using 24-dietary recall was underestimating the salt intake[49]. To overcome the limitation of the 24-hour dietary recall tool, the salt intake was also estimated through urinary salt excretion from 24-hour urine samples, a gold-standard method.

Nutritional status is mainly assessed through anthropometric measurements. Different anthropometric measures were used in this study to assess various nutritional aspects of the body, such as height and weight, to assess the individuals' BMI to categorise them into underweight, normal, and overweight categories. For BMI assessment, WHO growth reference charts for 5 to 19 years are not specific for Indian Adolescents, yet they are widely used.[23] BMI does not directly measure body fat and does not distinguish between muscle and body fat weight. BMI may overestimate the body fat in a person with a muscular build and may underestimate body fat in older adults who have lost muscle. Therefore, it was considered best to use BMI with other measures, such as WHR and MUAC.[50]

Waist and hip circumference are used to assess WHR, providing information regarding abdominal obesity.[50] MUAC is the body measurement least affected by weight and height changes compared to anthropometric measures such as BMI and WHR.[51] Therefore, MUAC was recorded to assess acute malnutrition among adolescents. However, MUAC fails to account for sex and age-specific differences as it is generally estimated through a single cut-off value.[52] The MUAC among the adolescents from the present study was on the lower end, signifying under-nutrition. The adolescents from the present study were from public schools, which usually cater

to the lower socio-economic strata of the population, where the proportion of starvation among adolescents is higher than the other SES groups. However, there is a lack of evidence-based MUAC cut-offs internationally. Due to this gap, several countries have established cut-offs that vary widely. Additionally, various skin fold thicknesses such as TSFT and SSFT were used to assess the subcutaneous fats.

Strengths and limitations

Before the final data collection, pilot testing was undertaken to assess the protocol's feasibility and the adequacy of the research instruments. Pilot testing helped identify problems that could have arisen later in data collection.[53] The study's strength was using validated and pretested tools in the Indian scenario and methodological data collection guidelines for the nutritional assessment.

Only public schools were enrolled in the study, which usually caters to the lower socio-economic groups in India. The implication of excluding private schools might lead to a lack of representation of participants across various socio-economic groups, limiting generalizability. But, the low-middle income group is a significant section of Indian society, and their representation in the study sample is one of the strengths of this study.

CONCLUSIONS

Adolescents from public schools of UT Chandigarh had poor nutritional status, with inadequate energy, fibre, iron, potassium, iodine, calcium, folate, vitamin A, vitamin B-12 and vitamin C intakes.

Schools should regularly check the nutritional status of the adolescents and may use the tools and measures used in the present study. However, in case of time constraints and lack of resources, schools may use only BMI or waist circumference as assessment criteria for regular monitoring of nutrition status among adolescents. Those identified as an underweight category through BMI may be assessed for their MUAC to know the severity of the undernourishment. In comparison, those falling in the overweight or obese categories of BMI may be assessed for WHR to determine the central obesity among adolescents. Mid-day meals should be regularly checked for their nutritional values and provided per the age requirements of the adolescents.

There is a need for behaviour change interventions to improve nutritional status in early life. Parents' and teachers' involvement can have far-reaching benefits regarding the sustainability of behavioural change for healthy dietary behaviours. The school-based intervention activities to improve nutrition among adolescents should be developed and implemented in a manner that can be replicated in other settings in India and low-income countries with modifications to account for differences in the contextual factors. The degree and type of activities must depend on the target populations' social and economic status and cultural aspects at a given time.

For further research on the effectiveness of policy-level interventions to reduce processed food consumption, policies such as food labels are required. In addition, the policymakers should test the implementation of the policy-level intervention in schools to ensure that only healthy foods are sold by replacing packaged and processed foods.

ACKNOWLEDGEMENT

We extend our special thanks to the George Institute of Global Health, Hyderabad, for providing us with the funds to conduct laboratory testing of the urine samples collected as part of this study.

REFERENCES

1. Beaglehole R, Epping-Jordan J, Patel V, Chopra M, Ebrahim S, Kidd M, et al. Improving the prevention and management of chronic disease in low-income and middle-income countries: a priority for primary health care. Lancet. 2008;372 (9642):940-9.
2. Teo K, Lear S, Islam S, Mony P, Dehghan M, Li W, et al. Prevalence of a healthy lifestyle among individuals with cardiovascular disease in high-middle-and low-income countries: The Prospective Urban Rural Epidemiology (PURE) study. JAMA. 2013 Apr 17;309(15):1613-21.
3. Gupta B, Gupta R, Sharma KK, Gupta A, Mahanta TG, Deedwania PC. Low prevalence of AHA-defined ideal cardiovascular health factors: A study of urban Indian men and women. Glob Heart. 2017;12(3):219-25.
4. National Non-comunicable Diseases Monitoring Survey (NNMS) 2017-18. Bangalore: Indian Council of Medical Research-National Centre for Disease Informatics and Research; 2020 [cited 2022 Jan 21]. Available from: https://www.ncdirindia.org/nnms/resources/Factsheet.pdf.
5. National Family Health Survey-5. Mumbai: International Institute of Population Sciences; 2021 [cited 2022 Jan 18]. Available from: http://rchiips.org/nfhs/NFHS-5_FCTS/India.pdf.
6. Diet, nutrition, and the prevention of chronic diseases: report of a joint WHO/FAO expert consultation. Geneva: World Health Organization; 2003 [cited 2021 Dec 23]. Available from: https://apps.who.int/iris/bitstream/handle/10665/42665/WHO_TRS_916.pdf.
7. Singh AK, Maheshwari A, Sharma N, Anand K. Lifestyle associated risk factors in adolescents. Indian J Pediatr. 2006 Oct;73(10):901-6.
8. Saraf D, Gupta S, Pandav C, Nongkinrih B, Kapoor S, Pradhan S, et al. Effectiveness of a school based intervention for prevention of non-communicable diseases in middle school children of rural North India: a randomized controlled trial. Indian J Pediatr. 2015 Apr;82(4):354-62.
9. Min J, Zhao Y, Slivka L, Wang Y. Double burden of diseases worldwide: coexistence of undernutrition and overnutrition-related non-communicable chronic diseases. Obes Rev. 2018 Jan;19(1):49-61.
10. Kaur S, Kaur M, Kumar R. Health promotion intervention to prevent risk factors of chronic diseases: Protocol for a cluster randomized controlled trial among adolescents in school settings of Chandigarh (India). PLoS One. 2022 Feb 17;17(2):e0263584.

11. Global School-Based Student Health Survey (Age 13-15). India: Central Board of Secondary Education; 2007 [cited 2021 Nov 16]. Available from: https://www.who.int/ncds/surveillance/gshs/2007_India_CBSE_fact_sheet.pdf.

12. Peltzer K, Pengpid S. Fruits and vegetables consumption and associated factors among in-school adolescents in five Southeast Asian countries. *Int J Environ Res Public Health.* 2012 Oct 11;9(10):3575-87. doi: 10.3390/ijerph9103575.

13. Kumar P, Kumar D, Ranjan A, Singh CM, Pandey S, Agarwal N. Prevalence of Hypertension and its Risk Factors Among School Going Adolescents of Patna, India. *J Clin Diagn Res*. 2017 Jan;11(1):SC01-SC04. doi: 10.7860/JCDR/2017/23886.9196.

14. Ha K, Chung S, Lee HS, et al. Association of Dietary Sugars and Sugar-Sweetened Beverage Intake with Obesity in Korean Children and Adolescents. *Nutrients*. 2016 Jan 8;8(1):31. doi: 10.3390/nu8010031.

15. National Survey on Drug Use and Health (NSDUH), 2013, (Age 12 or older for Asian population). North Carolina, USA: Separtment of Health and Human Services; [cited 2021 May 17]. Available from: https://www.samhsa.gov/data/data-we-collect/nsduh-national-survey-drug-use-and-health.

16. Jonnalagadda S, Mitchell D, Smiciklas-Wright H, Meaker K, Heel NV, Karmally W, et al. Accuracy of energy intake data estimated by a multiple-pass, 24-hour dietary recall technique. J Am Diet Assoc. 2000 Mar;100(3):303-8.

17. Hjartåker A, Andersen LF, Lund E. Comparison of diet measures from a food-frequency questionnaire with measures from repeated 24 hour dietary recalls. The Norwegian Women and Cancer Study. Public Health Nutr. 2007; 10(10):1094–103. https://doi.org/10.1017/S1368980007702872.

18. Bharathi AV, Kurpad AV, Thomas T, Yusuf S, Saraswathi G, Vaz M. Development of food frequency questionnaires and a nutrient database for the Prospective Urban and Rural Epidemiological (PURE) pilot study in South India: methodological issues. Asia Pac J Clin Nutr. 2008; 17(1):178–85. .

19. Sharma R. Revised Kuppuswamy's Socioeconomic Status Scale: Explained and Updated. Indian Pediatr. 2017 Oct 15;54(10):867-870.

20. Norton K, Whittingham N, Carter L, Kerr D, Gore C, Marfell-Jones M. Measurement techniques in anthropometry. Anthropometrica. 1996;1:25-75.

21. Miller V, Yusuf S, Chow CK, Dehghan M, Corsi DJ, Lock K, et al. Availability, affordability, and consumption of fruits and vegetables in 18 countries across income levels: findings from the Prospective Urban Rural Epidemiology (PURE) study. Lancet Glob Health. 2016;4(10):e695-703.

22. Nutrient Requirements for Indians. Hyderabad, India: Indian Council of Medical Research-National Institute of Nutrition; 2020 [cited 2021 Dec 3]. Available from: https://www.im4change.org/upload/files/RDA_short_report%281%29.pdf.

23. BMI-for-age (5-19 years). Geneva: World Health Organization; 2007 [cited 2022 Feb 21]. Available from: https://www.who.int/tools/growth-reference-data-for-5to19-years/indicators/bmi-for-age.

24. World Health Organization. Waist circumference and waist-hip ratio: report of a WHO expert consultation, Geneva, 8-11 December 2008. https://apps.who.int/iris/bitstream/handle/10665/44583/?sequence=1.

25. Khadilkar A, Mandlik R, Chiplonkar S, Khadilkar V, Ekbote V, Patwardhan V. Reference centile curves for triceps skinfold thickness for Indian children aged 5–17 years and cut-offs for predicting risk of childhood hypertension: a multi-centric study. Indian Pediatr. 2015 Aug;52(8):675-80.

26. Brosius F. Statistical Package for the Social Sciences 21. IBM Corp; 2013 [cited 2021 Aug 15]. Available from: https://www.sussex.ac.uk/its/pdfs/SPSS_Brief_ Guide_21.pdf.

27. Lucko AM, Doktorchik C, Woodward M, Cogswell M, Neal B, Rabi D, et al. Percentage of ingested sodium excreted in 24-hour urine collections: A systematic review and meta-analysis. J Clin Hypertens (Greenwich). 2018 Sep;20(9):1220-1229.

28. Food and Nutrition Technical Assistance. Washington, DC: USAID; 2013 [cited 2022 Feb 21]. Available from: https://www.fantaproject.org/sites/default/files/resources/MUAC%20Systematic%20Review%20_Nov%2019.pdf.

29. Bwalya BB. Nutritional status among female adolescents aged (15–19 years) in Zambia: why it matters. Horizon J Med and Med Sci. 2015;1(1):1-7.

30. Parimalavalli R, Sangeetha M. Anthropometric measurements and nutrient intake of adolescent girls. Anthropologist. 2011 Apr 1;13(2):111-5.

31. Nicholaus C, Martin HD, Kassim N, Matemu AO, Kimiywe J. Dietary Practices, Nutrient Adequacy, and Nutrition Status among Adolescents in Boarding High Schools in the Kilimanjaro Region, Tanzania. J Nutr Metab. 2020;3592813.

32. Nago ES, Lachat CK, Huybregts L, Roberfroid D, Dossa RA, Kolsteren PW. Food, energy and macronutrient contribution of out-of-home foods in school-going adolescents in Cotonou, Benin Br J Nutr. 2010 Jan;103(2):281-8.

33. Saxena NC. Hunger, under-nutrition and food security in India. In poverty, chronic poverty and poverty dynamics. Springer Sci Rev. 2018;(pp. 55-92).

34. Iglesia I, Doets EL, Bel-Serrat S, Román B, Hermoso M, Quintana LP, et al. Physiological and public health basis for assessing micronutrient requirements in children and adolescents. Matern Child Nutr. 2010;2:84-99.

35. Pal A, Pari AK, Sinha A, Dhara PC. Prevalence of undernutrition and associated factors: A cross-sectional study among rural adolescents in West Bengal, India. Int J Pediatr Adolesc Med. 2017 Mar;4(1):9-18.

36. Ng N, Stenlund H, Bonita R, Hakimi M, Wall S, Weinehall L. Preventable risk factors for noncommunicable diseases in rural Indonesia: prevalence study using WHO STEPS approach. Bull World Health Organ. 2006;84(4):305-13.

37. Jagadesan S, Harish R, Miranda P, Unnikrishnan R, Anjana RM, Mohan V. Prevalence of overweight and obesity among school children and adolescents in Chennai. Indian Pediatr. 2014 Jul;51(7):544-9.

38. Jain S, Pant B, Chopra H, Tiwari R. Obesity among adolescents of affluent public schools in Meerut. Indian J Public Health. 2010 Jul-Sep;54(3):158-60.

39. Goyal JP, Kumar N, Parmar I, Shah VB, Patel B. Determinants of Overweight and Obesity in Affluent Adolescent in Surat City, South Gujarat region, India. Indian J Community Med. 2011;36(4):296-300.

40. Praveen G, Subhashini K. Assessment of the Nutritional Status and Determinants of Malnutrition among School Going Adolescents in the Rural Field Practice Area of the Medical College, Hassan, Karnataka. Int J Trop Dis Health. 2019;40(2):1-13.

41. Stanfield P, Stanfield P, Hui Y. Nutrition and diet therapy: self-instructional modules. ADA, editor. USA: Jones & Bartlett Learning; 2003. https://pdfgoal.com/downloads/nutrition_and_diet_therapy_self_instructional_modules.

42. de Onis M, Dasgupta P, Saha S, Sengupta D, Blössner M. The National Center for Health Statistics reference and the growth of Indian adolescent boys. Am J Clin Nutr. 2001 Aug;74(2):248-53.

43. Ricardo G, Caldeira G, Catarina A. Prevalence of overweight and obesity and adiposity central indexes among school-aged children in Santa Catarina. Rev Bras Epidemiol. 2009;12(3):1-12.

44. Deurenberg P, Pieters JJ, Hautvast JG. The assessment of the body fat percentage by skinfold thickness measurements in childhood and young adolescence. Br J Nutr. 1990 Mar;63(2):293-303.

45. Agarwal KN, Saxena A, Bansal AK, Agarwal DK. Physical growth assessment in adolescence. Indian Pediatr. 2001 Nov;38(11):1217-35.

46. Tang HK, Bowe SJ, Nguyen THHD, Dibley MJ. Triceps and subscapular skinfold thickness percentiles of a school-based sample of adolescents in Ho Chi Minh City, Vietnam. Eur J Clin Nutr. 2020 Oct;74(10):1483-1487.

47. Hastuti J, Rahmawati NT, Suriyanto RA, Wibowo T, Nurani N, Julia M. Patterns of Body Mass Index, Percentage Body Fat, and Skinfold Thicknesses in 7- to 18-Year-Old Children and Adolescents from Indonesia. Int J Prev Med. 2020 Aug 19;11:129.

48. Dong J, Zhao X, Dong H, Yan Y, Mi J. Can spot urine measurement be a substitute for 24-hour urine measurement to estimate sodium intake in adolescents? *Pediatr Investig* 2021; **5**(1): 12-20.

49. Cogswell M, Zhang Z, Carriquiry A, et al. Sodium and potassium intakes among US adults: NHANES 2003-2008. Am J Clin Nutr. 2012 Sep;96(3):647-57.

50. Cashin K, Oot L. Practical Tool for Program Planners, Managers, and Implementers (Guide to Anthropometry). Washington, DC: Food and Nutrition Technical Assistance; 2018 [cited 2021 Nov 24]. Available from: https://www.fantaproject.org/sites/default/files/resources/FANTA-Anthropometry-Guide-May2018.pdf.

51. Singhal N, Misra A, Shah P, Gulati S. Effects of controlled school-based multi-component model of nutrition and lifestyle interventions on behavior modification, anthropometry and metabolic risk profile of urban Asian Indian adolescents in North India. Eur J Clin Nutr. 2010 Apr;64(4):364-73.

52. Madden AM, Smith S. Body composition and morphological assessment of nutritional status in adults: a review of anthropometric variables. J Hum Nutr Diet. 2016 Feb;29(1):7-25.

53. Etchegaray JM, Fischer WG. Understanding evidence-based research methods: pilot testing surveys. HERD. 2011;4(4):143-7.

Figure Click here to access/download;Figure;Figures.docx

Nutritional Assessment of Adolescents: a Cross-sectional Study from Public Schools of North India

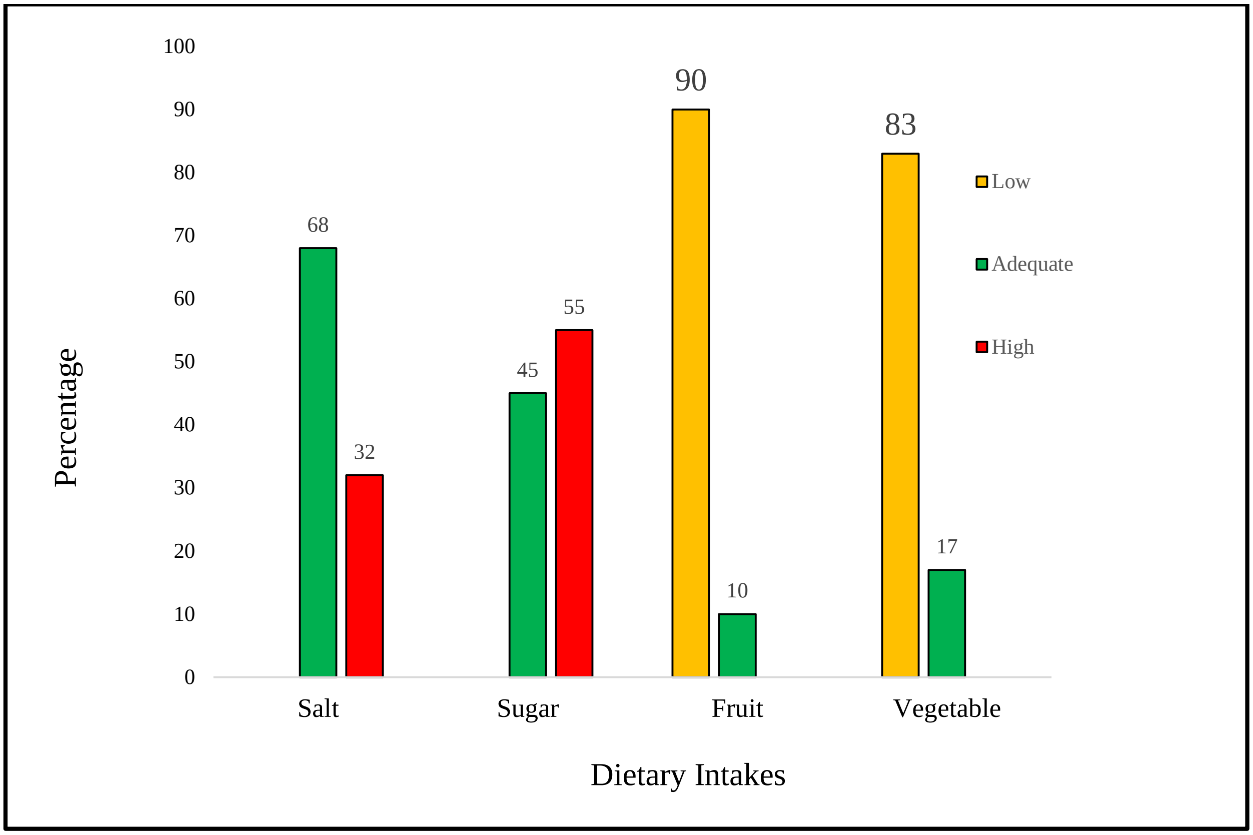

Cut-offs for dietary intakes| adequate salt: ≤ 5 g/d, high salt: > 5 g/d, adequate sugar: ≤ 20 g/d for girls and 25 g/d for boys, high sugar: > 20 g/d for girls and 25 g/d for boys, low fruit < 100g/d, adequate fruit: ≥ 100g/d, low vegetable < 300g/d and adequate vegetable: ≥ 300g/d.

Figure 1. Adequacy of dietary intake of salt, sugar, fruit and vegetables among adolescents (n=453)

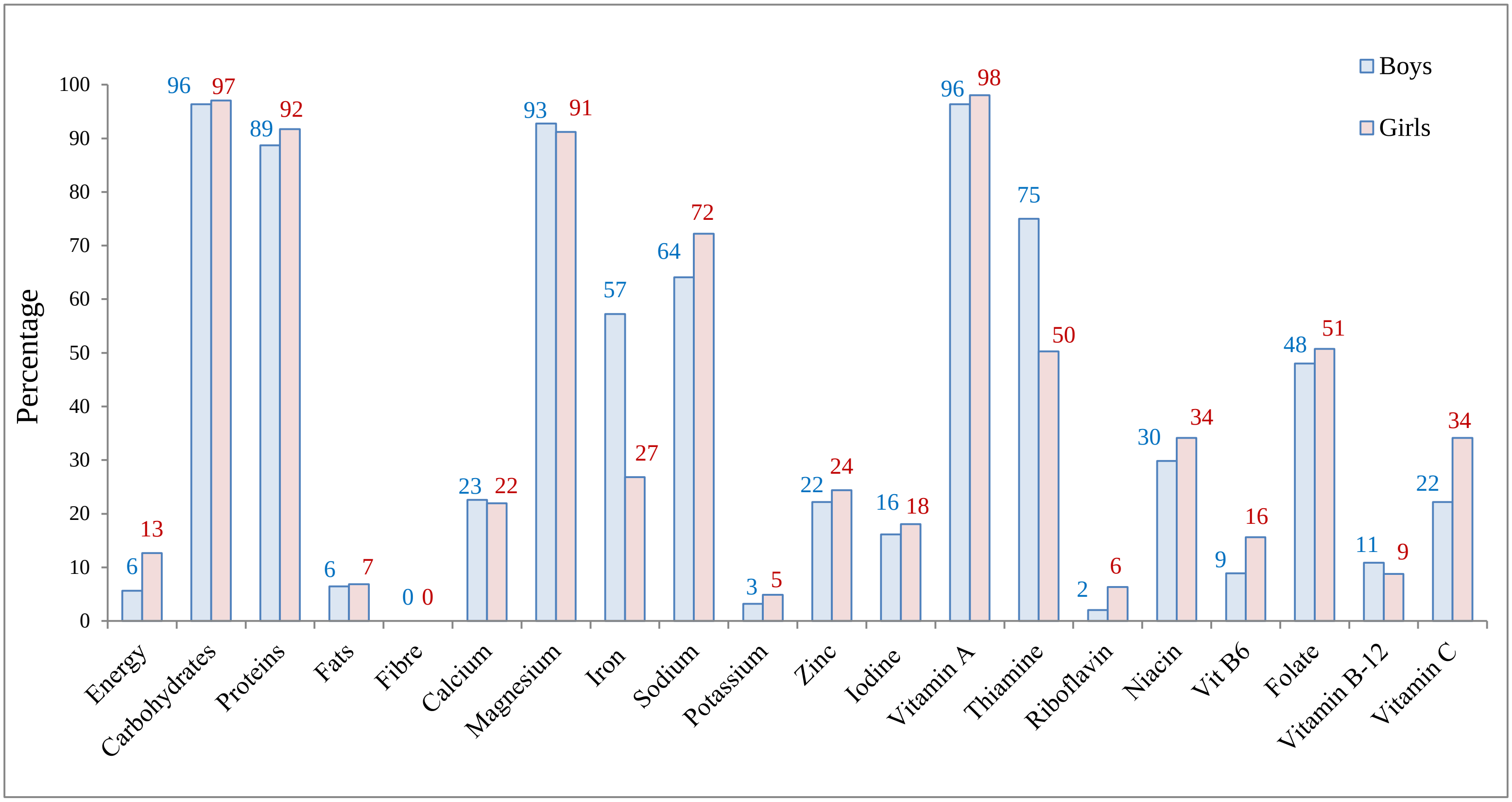

Cut-offs for all macronutrients, minerals and vitamins intake depicted in the figure were adopted from Recommended Dietary Allowance (RDA) for the Indian populations from the Indian Council of Medical Research-National Institute of Nutrition (ICMR-NIN), Hyderabad (2020)[22]

Figure 2: Proportions of adolescents with adequate intake of nutrients (n=453)

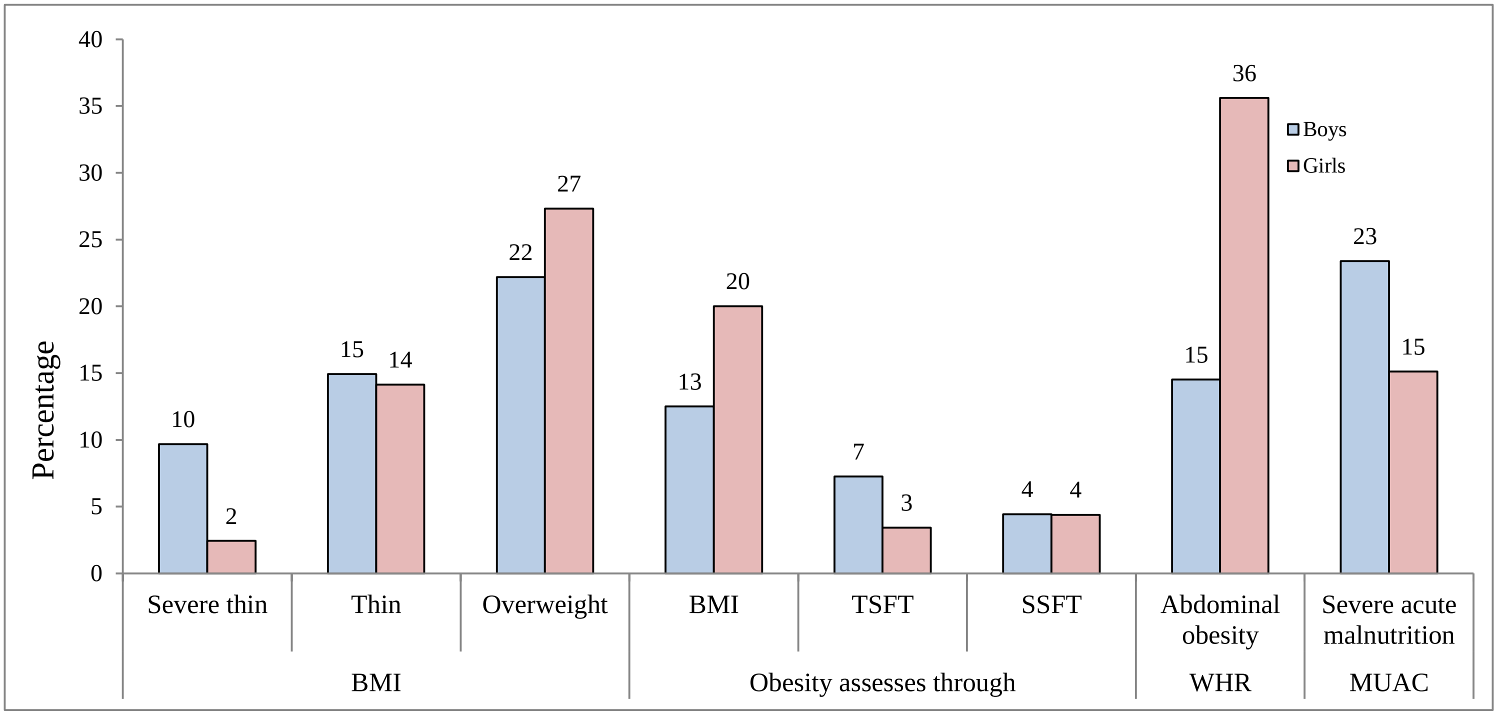

Cut-offs for malnourishment assessment among adolescents| Body Mass Index (BMI): adolescents (WHO growth reference percentiles- age and gender specific for 5-19 years, 2007) = ≤ -2SD is severe thinness, =-2SD to -1SD is thinness, -1SD to +1 SD is normal, +1 SD to + 2 SD is overweight and above + 2 SD is obese
Triceps skinfold thickness (TSFT) and sub-scapular skinfold thickness (SSFT): Reference curves of Indian adolescents (5-17 years) were used
Abdominal Obesity assessed through Waist-Hip Ratio (WHR)= 0.9 for boys and 0.80 for girls
Mid-upper Arm Circumference (MUAC)= ≥ 23 cm for boys and ≥ 22 cm for girls

Figure 3: Malnourishment assessed through anthropometric measures

Nutritional assessment of adolescents: a cross-sectional study from public schools of North India

Table 1: Socio-demographic Characteristics of the Participating Adolescents (n=453)

Characteristics		n (%)
Sex		
	Male	248 (55)
	Female	205 (45)
Age (years)		
	10-12	82 (18)
	13-15	375 (81)
	16-18	5 (1)
Religion		
	Hindu	395 (87)
	Muslim	17 (4)
	Sikh	37 (8)
	Others (Christian, Jain, Buddhist)	4 (1)
Caste		
	General	299 (66)
	Other backward class (OBC)	45 (10)
	Scheduled caste (SC)	109 (24)
	Scheduled tribe (ST)	0
Socio-economic status of household		
	Lower	39 (8.6)
	Upper Lower	277 (61)
	Lower Middle	107 (24.2)
	Upper Middle	29 (6)
	Upper	1 (0.2)

Table 2: Macro-Nutrient Intake among Adolescents

	Mean (SD): (n=453)									
**Characteristics**	Energy (Kcal/d)	P	Carbohydrates (g/d)	P	Protein (g/d)	P	Total fat (g/d)	P	Fibre (g/d)	P
Overall	1813 (594)		294 (88[illegible]		54 (18)		47 (26)		7 (3)	
Sex										
Male	1838 (570)	0.3	297 (83)	0.4	56 (18)	0.009	47 (26)	0.7	8 (3)	0.05
Female	1782 (622)		290 (94)		51 (17)		46 (26)		7 (3)	
Age (years)										
10-12	1738 (516)		278 (72)		52 (19)		46 (23)		7 (3)	
13-15	1829 (602)	0.5	297 (90)	0.2	54 (17)	0.7	47 (26)	0.9	7 (3)	0.5
16-18	1852 (1109)		298 (150)		53 (29)		50 (46)		8 (4)	

Cut-offs for all macronutrients, minerals and vitamins intake depicted in the figure were adopted from Recommended Dietary Allowance (RDA) for the Indian populations from the Indian Council of Medical Research-National Institute of Nutrition (ICMR-NIN), Hyderabad (2020)[22]

Table 3: Mineral Intake among Adolescents

	Mean (SD): (n=453)													
**Characteristics**	Calcium (mg/d)	P	Magnesium (mg/d)	P	Iron (mg/d)	P	Sodium (mg/d)	P	Potassium (mg/d)	P	Zinc (mg/d)	P	Iodine (µg/d)	P
Overall	626 (257)		472 (148)		15 (5)		1815 (751)		1954 (731)		9 (3)		67 (31)	
Sex														
Male	628 (247)	0.9	488 (154)	0.01	16 (5)	0.002	1860 (776)	0.2	1961 (660)	0.9	9 (3)	0.009	66 (30)	0.5
Female	623 (270)		453 (137)		14 (5)		1760 (716)		1945 (810)		8 (2)		68 (32)	
Age (years)														
10-12	622 (268)		458 (142)		14 (5)		1693 (788)		1865 (627)		8 (3)		65 (28)	
13-15	628 (256)	0.9	475 (148)	0.6	15 (5)	0.2	1838 (739)	0.2	1974 (746)	0.5	9 (3)	0.7	67 (32)	0.9
16-18	565 (214)		462 (243)		14 (8)		2043 (972)		1904 (1149)		8 (4)		65 (24)	

Cut-offs for all macronutrients, minerals and vitamins intake depicted in the figure were adopted from Recommended Dietary Allowance (RDA) for the Indian populations from the Indian Council of Medical Research-National Institute of Nutrition (ICMR-NIN), Hyderabad (2020)[22]

Table 4: Vitamin Intake among Adolescents

	Mean (SD): (n=453)															
**Characteristics**	Vit A (µg/d)	P	Thiamine (mg/d)	P	Riboflavin (mg/d)	P	Niacin (mg/d)	P	Vit B6 (mg/d)	P	Folate (µg/d)	P	Vit B 12 (µg/d)	p	Vit C (mg/d)	P
Overall	2497 (2526)		1.4 (0.5)		1.1 (0.4)		13 (4.7)		1.4 (0.5)		248 (106)		1.2 (0.8)		54 (49)	
Sex																
Male	2473 (2671)	0.8	1.5 (0.5)	0.001	1.1 (0.4)	0.5	14 (4.9)	0.005	1.5 (0.5)	0.02	248 (106)	0.04	1.1 (0.8)	0.6	50 (41)	0.03
Female	2526 (2345)		1.3 (0.5)		1.08 (0.4)		12 (4)		1.4 (0.4)		227 (105)		1.2 (0.9)		59 (57)	
Age (years)																
10-12	2567 (2978)		1.4 (0.5)		1.1 (0.4)		12.4 (4.7)		1.3 (0.4)		225 (93)		1.2 (0.7)		48 (41)	
13-15	2459 (2411)	0.3	1.4 (0.5)	0.5	1.1 (0.4)	0.7	13.1 (4.6)	0.5	1.4 (0.5)	0.4	241 (108)	0.4	1.2 (0.8)	0.8	55 (51)	0.4
16-18	4172 (2980)		1.3 (0.8)		0.9 (0.5)		13.1 (9)		1.4 (0.9)		249 (119)		0.9 (0.3)		64 (46)	

Cut-offs for all macronutrients, minerals and vitamins intake depicted in the figure were adopted from Recommended Dietary Allowance (RDA) for the Indian populations from the Indian Council of Medical Research-National Institute of Nutrition (ICMR-NIN), Hyderabad (2020)[22]

Click here to access/download
Supporting Information
Study_protocol.pdf

Click here to access/download
Supporting Information
Supplement.docx

STROBE checklist

Click here to access/download

Supporting Information

STROBE_checklist.pdf
