# Supplementary material for: Nutritional assessment of adolescents: A cross-sectional study from public schools of North India
Source: PLoS One. 2025 Jan 14;20(1):e0316435. doi: 10.1371/journal.pone.0316435 (PMC11731870; doi:10.1371/journal.pone.0316435)
Supplement: Attachment — Submitted filename: Responses to the comments.docx [file pone.0316435.s005.docx]

**RESPONSE TO REVIEWER'S COMMENTS**

**Response to Reviewer 1**

We thank the reviewer for the comments, which helped us improve the paper. Further, in light of valuable comments from the reviewer, we have revised the manuscript by addressing all comments. The response to each comment is provided in blue, and the manuscript changes are highlighted using the 'track changes' command.

1. *In this result section, need not write the need of health promotion interventions*

Thanks for your input. As suggested, we have removed this statement.

2. *Mention the % of energy intake from carbohydrate referring to dietary goals . Include in table 2 the reference standards-dietary goal %*

Thank you. Based on your suggestion, we have included all the reference standards used for macro and micronutrients in the supplement as appendix S9. We have also included these in the text in the lines 207-215.

3. *Please include all RDA in the table 2 in brackets*

We thank the reviewer for this valuable suggestion. However, as the table was too difficult to comprehend with the addition of RDA values in the brackets, we have provided RDA in the supplement as separate tables in Appendix S9.

4. *To state adolescents are deficient in macro and micronutrients , RDA should be mentioned in the text and table*

We agree and have provided these details in the manuscript as figure 2, as tables in tables S4, S5 and S6, and at various places in the text from lines 189-249.

5. *Create 95% confidence interval to see whether the cut-off is included within the CI to say it is normal or less*

Thank you for highlighting this point. Based on the comments, we have added the 95% CI in lines 251-255.

6. *What does the authors mean by normal abdominal obesity?*

We thank the reviewer for providing an opportunity for clarification. With 'normal abdominal obesity, ' authors meant the waist-hip ratio that falls within the normal range, which is the waist-hip ratio of ≤ 0.9 for males and ≤ 0.85 for females. However, we agree that the term may confuse the readers. Therefore, we have changed it to 'no abdominal obesity'.

7. *Optimal MUAC cut off point is different for different age groups in your study (10-16 yrs) . Cannot take an average*

Thank you! We want to clarify that mid- upper arm circumference (MUAC) depicts severe malnourishment. We agree that MUAC measurements can vary widely for adolescents based on factors like growth spurts, gender, and individual body composition. However, there are no universally accepted cut-offs in this age group. Moreover, a single cut-off point of 23 cm for males and 22 cm for females is widely used. We have added this limitation of MUAC in the discussion from lines 360-370, supported by reference 52 in the manuscript.

8. *Clearly mention in this table what each value represents. What is 88? Is it SD? What is 294? Is it mean or median? Label it.*

Thanks to the reviewer, we would like to clarify that we have added these details at the top of the table. The values provided are in Mean (SD).
