# Supplementary material for: Nutritional assessment of adolescents: A cross-sectional study from public schools of North India
Source: PLoS One. 2025 Jan 14;20(1):e0316435. doi: 10.1371/journal.pone.0316435 (PMC11731870; doi:10.1371/journal.pone.0316435)
Supplement: Attachment — Submitted filename: Response to reviewer.docx [file pone.0316435.s006.docx]

**RESPONSE TO REVIEWER'S COMMENTS**

**Response to Reviewer 2**

We thank the reviewer for the comments, which helped us improve the paper. Further, in light of valuable comments from the reviewer, we have revised the manuscript by addressing all comments. The response to each comment is provided in blue, and the manuscript changes are highlighted using the 'track changes' command.

1. *An abstract needs to be abridged and made crisp after addition of more information. It needs to add definitions/cut-offs for the various parameters used. How were the dietary history converted to micronutrient intakes etc. Might want to restrict to key parameters.*

We thank the reviewer for the input. Based on this we have added more details in the abstract.

2. *Not sure why it is recommended that schools should regularly assess adolescent nutritional status. I am sure it will also apply for children. So are we saying that all schools should have this process as a part of regular work/duties and what kind of intervention is to be done by them. Or this is a part of the school health program of the health system.*

We appreciate the reviewer providing us with the opportunity to clarify this important aspect of our study. The literature indicates that behavioural risk factors, such as unbalanced diets, begin to emerge in early adolescence. These risk factors are known to lead to chronic diseases later in life. In low- and middle-income countries like India, there is a double burden of malnutrition, with a significant proportion of adolescents being either underweight or overweight.

Schools are critical environments where children spend most of their day and learn from their teachers and peers. Therefore, schools can be ideal settings to inculcate healthy behaviours early in life. Currently, in schools, there is only one chapter in the sixth-grade textbook covering the sources, components, and importance of nutrition. However, there is no specific chapter on this crucial aspect of health until the tenth grade.

Our study was part of a larger Cluster Randomized Trial (referred to in the manuscript as number 11) aimed at assessing the impact of a health promotion intervention package on reducing behavioural risk factors related to chronic diseases. One component of the intervention focused on dietary behavioural risk factors, such as high salt and sugar intake and low fruit and vegetable intake.

Despite the challenges posed by the COVID-19 pandemic, our study was effective in reducing some of these behavioural risk factors. We propose that future studies build on this work to develop a module that can be easily integrated into the school curriculum. This would promote healthy behaviours from early life, potentially reducing the ever-increasing burden of chronic diseases in the future.

3. *Introduction: The last line should read public schools in a city in North India.*

Thank you, we have corrected the sentence as suggested.

4. *A national level adolescent survey for NCD risk factors has been conducted as a part of the NNMS, which has not been referred to at all. Why? Mathur P. etal. Baseline risk factor prevalence among adolescents aged 15-17 years old: findings from NNMS of India. BMJ Open. 2021 Jun 29;11(6):e044066.*

We thank the reviewer for bringing this to our attention. While we did refer to NNMS (reference number 4) in our manuscript, we have now included the additional reference (number 5) based on the reviewer’s suggestion.

5. *Methods:….. 106 public schools had 8th grade classes and “on average 10790 adolescents study in the 8th grade. Not clear – average per school or is it total and not average?*

We would like to clarify that data collected from the Department of School Education Chandigarh revealed that 106 public schools had eighth-grade classes, and on average 10,790 adolescents study in 8th grade in these schools. Most schools had four to five sections in 8th grade class. Each section of the class had about 30 adolescents.

6. *Sampling of schools and classes and adolescents is not well described. If the sample size was 360 , why were 462 sampled?*

We thank the reviewer for providing us with the opportunity to address this crucial aspect of our study. Literature suggests that the behavioural risk factors of chronic diseases start to emerge during early adolescence. Therefore, based on this knowledge and analysis of the school curriculum in UT Chandigarh, adolescents from 8th grade studying in public schools were selected for this study.

A total of twelve clusters (schools) were sampled based on the formula mentioned below.

c = 1 + f [ π0 (1 - π0)/m + π1(1 - π1)/m + k2 (π0 2 + π1 2)] / (π0 - π1)2

Where c is the number of clusters required, f = 7.84 for 5% type I error and 80% power, π1 is the expected proportion of the behavioural risk factor in the intervention arm, π0 is the expected proportion of risk factor in the comparison arm, m is the number of individuals in each cluster (assumed equal in all clusters), and k is the coefficient of variation in the (true) proportions between clusters in each study arm.

Data collected from the Department of School Education Chandigarh revealed that 106 public schools had eighth-grade classes, and on average 10,790 adolescents study in 8th grade in these schools. Most schools had four to five sections in 8th grade class. Each section of the class had about 30 adolescents. Therefore, the cluster size (m) was 30 adolescents in each section.

The c value for each primary outcome was calculated separately using data for adolescents from recent studies. Out of all risk factors, the maximum c value (12) was considered the sample size of the number of clusters for the study, i.e., sugar intake. Thus, a sample size of 360 adolescents (12 clusters x 30 adolescents per cluster) was estimated to provide valid statistical inference about the impact of the intervention.

However, in all twelve consenting schools, the eligible eighth-grade adolescents were 462. All were included in the study owing to ethical considerations. The details of the sampling and sample size are provided in the protocol paper of the study which has been referred to in the manuscript.

7. *Not clear why is analysis of school curriculum is referred to?*

Analysis of school curriculum was referred to as the present study was part of a larger cluster randomized controlled trial to assess the impact of health promotion intervention. It was important to incorporate a health promotion intervention package in the school settings without disturbing the school curriculum for easy replication of the study for sustainable behaviours.

8. *Primary outcome variable has been labeled as Nutritional status. It is not clear what does this status refer to (anthropometry, dietary intake?)*

Thank you! We have corrected it to ‘Dietary Intake’.

9. *Urinary salt excretion is a reasonably good measure of population level sodium intake and not at individual level. A single measurement is not appropriate for estimation of salt intake at individual level. Details of sodium assessment including the formula used should be provided.*

We acknowledge the reviewer's point regarding urinary salt excretion as a measure more suited for assessing population-level sodium intake rather than individual-level intake. However, it's important to note that urinary sodium excretion is widely regarded as the 'gold-standard' method for analyzing sodium levels. Our decision to include this measure in the study alongside dietary history was aimed at mitigating the limitations of 24-hour dietary recalls, which tend to underestimate salt levels.

Moreover, urinary excretion provides an objective parameter that necessitates laboratory testing, thus enhancing the accuracy of assessing salt levels.

10. *Reference 27 seems inappropriate to define cut-off at individual level for sodium intake.*

Thank you, the paper is a systematic review which provided cut-offs from urinary sodium at 3.3.4. section of the referred paper.

11. *PURE study software should have a reference.*

Added it as a reference in the manuscript with number 20.

12. *Reference to senior faculty member and DC members should be deleted.*

Thank you for the input. We have deleted it.

13. *The cut-offs for Waist circumference and MUAC seem to be for adults. Are these adolescent-specific?*

Waist-circumference and MUAC have single-point cut-offs. These are for adolescents. The reference for this has been provided as numbers 26 and 27.

14. *The need for p-values is not justified given that there were no hypotheses to begin with.*

Thank you, we have added a hypothesis for the study in the methods.

15. *Results:Table 1 can be deleted and matter in the text is sufficient. At best can be kept as a supplementary table.*
    Thank you. Based on the reviewer’s suggestion we have shifted Table 1 to the supplement as Appendix 8: Table S1.
16. *While in the results fat intake is classified as slightly below RDA (provides 25% of energy) in the Figure 2, only 6-7% have adequate intake? Seems to be a discrepancy.*

Thank you for your question. However, we'd like to clarify that while the fat intake may be slightly below the Recommended Dietary Allowance (RDA), it exhibits a consistent pattern across all age and sex categories. Upon re-evaluating the results, we found no discrepancy, with only 6% of boys and 7% of girls demonstrating adequate fat intake.

17. *I have reservations about the use of theoretical levels of micronutrients to estimate their intake as we know these vary based on the soil composition and manure. There is too much emphasis on micronutrients in the paper. In the absence of biochemical estimation even in a subsample, I would not focus on them.*

We appreciate the input from the reviewer and would like to clarify our methodology for estimating micronutrients. To estimate both micro and macronutrients, such as protein, carbohydrates, fat, vitamins, and minerals, we utilized the PURE study computer software. This software has been specifically developed for assessing the intake of Indian foods and has previously been validated in Indian settings.

The tool encompasses a comprehensive list of 143 different food items commonly consumed in India. Upon entering a participant's average portion size and frequency of food consumption per day into the software, it can estimate 72 different macro and micronutrients for that individual.

Our approach involved inputting participants' dietary data, including portion sizes and consumption frequency, into the software. Based on the Indian dietary data provided by the ICMR-NIN (Indian Council of Medical Research-National Institute of Nutrition), the software employs the weight of raw ingredients in the food items. The NIN furnishes nutrient data per 100 grams of raw food ingredients. Therefore, the software utilizes this data in proportion to the quantity of ingredients used to prepare the food items.

In our methodology, the software estimates the quantity of nutrients based on the raw ingredients typically used in food preparation. This allows for the estimation of various macro and micronutrients present in the raw ingredients of the consumed food items.

While dietary assessment provides valuable insights, we agree that it may not be as accurate as biochemical assessment for micronutrient evaluation. For additional clarity on our methodology, please refer to Appendix S3 and S4 included in the supplement.

Some of the earlier published studies have used the same dietary tool to assess micro and macronutrients.

1. Kaur J, Kaur M, Chakrapani V, Webster J, Santos JA, Kumar R. Effectiveness of information technology-enabled 'SMART Eating' health promotion intervention: A cluster randomized controlled trial. PLoS One. 2020 Jan 10;15(1):e0225892. doi: 10.1371/journal.pone.0225892.
2. Mahajan R, Malik M, Bharathi AV, Lakshmi PV, Patro BK, Rana SK, Kumar R. Reproducibility and validity of a quantitative food frequency questionnaire in an urban and rural area of northern India. Natl Med J India. 2013 Sep-Oct;26(5):266-72.
18. *Figure 1 The use of Nar chart is inappropriate as it totals to 100%. Pie or a stacked bar chart is a better alternative.*

We express our gratitude to the reviewer for their valuable suggestion. Following their advice, we have made the necessary adjustments to the figure, now presenting it in a stacked bar format.

19. *Figure 3. it says severe acute malnutrition by MUAC and the cut off in legend is MUAC> 23/22 cms!!*

Yes, the figure shows severe acute malnutrition by MUAC separately for boys and girls. The cut-off point of MUAC for adolescent boys is ≥ 23cm and for girls is ≥ 22cm. So, the figure depicts that out of all boys recruited 23% were severely malnourished, i.e., 23% of boys had MUAC equal to or below 23 cm and similarly 15% of girls had MUAC equal to or below 22cm.
