# Supplementary material for: Nutritional assessment of adolescents: A cross-sectional study from public schools of North India
Source: PLoS One. 2025 Jan 14;20(1):e0316435. doi: 10.1371/journal.pone.0316435 (PMC11731870; doi:10.1371/journal.pone.0316435)
Supplement: Attachment — Submitted filename: Response to reviewer_09122024.docx [file pone.0316435.s007.docx]

**RESPONSE TO REVIEWERS COMMENTS**

**Reviewer 3**

It is a very relevant study and has the potential to provide useful information

We thank the reviewer for the comments, which helped us improve the paper. Further, in light of valuable comments from the reviewer, we have revised the manuscript by addressing all comments. The response to each comment is provided in blue, and the manuscript changes are highlighted using the 'track changes' command.

1. *Line 173 can be re-structured to make it more comprehensible (32% and 55% of salt and sugar line)*

Thank you! Based on the reviewer’s suggestion, we have revised the line to make it more concise and clear.

2. *MUAC is an unusual metric to assess adolescents because of variable growth spurts - it as shown an unusual proportion of 'Severe acute malnutrition' which is not corroborated by the BMI values.*

We would like to thank the reviewer for providing the opportunity to clarify this important point. As highlighted in the discussion section, MUAC is the anthropometric measure least affected by changes in weight and height, compared to other measures such as BMI and Waist-Hip Ratio (WHR). For this reason, MUAC was selected to assess acute malnutrition among adolescents, in addition to BMI, which is more sensitive to the fluctuations in weight and height that are common during adolescence.

3. *Reference 30 for MUAC does not state that less than 22/23 is malnutrition and definitely not severe malnutrition. SO figure 3 is a misrepresentation*

We would like to express our gratitude to the reviewer for their valuable feedback and take this opportunity to clarify a few points regarding reference 30. In Section 3.1.1 of this reference, a MUAC cut-off of 22 cm for adolescents is provided. Additionally, Figure 2 in the same reference summarizes the MUAC cut-offs for adolescents, adults, and pregnant women, based on existing literature. As mentioned in lines 332-334 of the discussion section, there is a recognized lack of internationally established, evidence-based MUAC cut-offs, which has resulted in variations in the thresholds used across different countries. In light of this gap, we have adopted the MUAC cut-off presented in reference 30 for the current study. Furthermore, we would like to clarify that MUAC, as a single cut-off measure, is commonly used to assess severe acute malnutrition.

4. *Lines 326 to 335 seem redundant. If the authors say that MUAC is not ideal and also state that because it is a public school and the students are anyways starving (line 331) then that makes the null hypothesis irrelevant. SO the basic premise of the study is debunked - suggesting that this is an ad hoc analysis of existing data.*

Thank you! We would like to take this opportunity to clarify that in lines 326-335, we are conveying that MUAC was used alongside other assessment measures, such as BMI and WHR. MUAC is less affected by changes in weight and height, which is particularly important during adolescence, a growth phase where BMI and WHR alone may not fully capture nutritional status. We aimed for a more holistic approach by incorporating MUAC, a measure that we believe is robust despite its limitations. We did not state that MUAC is not ideal; rather, we acknowledged its potential limitations, including its use as a single cut-off measure without a universally accepted threshold.

These points in the manuscript are discussed in the context of our findings, where we report that 23% of boys and 15% of girls in our study were severely malnourished. We attribute these high figures to the fact that our participants were from public schools, which primarily serve lower socioeconomic groups, where access to nutritious food is often limited.

5. *The conclusions and recommendations do not align with the results. This was not a health systems/policy research/analysis to suggest systemic changes. This is an observational study which can provide suggestions for further studies.'*

We appreciate the reviewer’s feedback. We acknowledge that our study is an observational study rather than a health systems or policy analysis. The conclusions and recommendations provided were intended to highlight key findings from the study and suggest areas for further research. Our conclusions emphasise the need for further and regular investigations into the nutritional status of adolescents. Given that our study reveals a high burden of malnutrition among adolescents, we also recommend that future research focus on interventions aimed at promoting healthy dietary behaviours to address this ongoing issue. Howeever, based on the reviewer’s comment we have revised the section to ensure that the recommendations are intended to guide future research in this area.

6. *The p values mentioned in the tables - what statistical tests were used? Were non parametric tests used for relevant variables?*

Thank you! In our study, the data were normally distributed, and therefore, parametric tests such as the t-test and ANOVA were used to calculate the p-values reported in the tables. No non-parametric tests were applied, as the assumptions of normality were met for the relevant variables. We have ensured that the statistical tests used are clearly outlined in the lines 134-143.

**Reviewer 4:**

Thank you for the opportunity. The study provides a good insight into nutrition among adolescents attending public schools in north India.

We thank the reviewer for their time to review the manuscript. We agree to all the issues that have been raised. The response to each of the comments is provided in blue colour. The changes have been made in the manuscript using the ‘track changes’ command.

1. *The introduction, methodology and discussion can be more concise. For example, the PURE software is explained in both the data collection and data management sections. There is no need to include investigator initials in the methodology and should be restricted to the author contribution section. A large part of the discussion seems to be reiterating results. Rather, the implications of the findings, and comparisons should be on focus. The discussion seems to focus on the proportion of risk factors while the results section mostly provides the mean and standard deviation. The strengths and limitations section does not mention specific strengths or problems identified in pilot testing. Some limitations seem to be mentioned before this section starts. This should be organized better.*

We thank the reviewer for their valuable input. In response, we have revised the introduction, methods, and discussion sections to make them more concise, incorporating the suggestions provided. The discussion section has been further refined to emphasise the implications of the findings and facilitate comparisons. Additionally, we have reorganised these sections to improve the overall flow. We would also like to clarify that the proportions mentioned in the discussion section are derived from Figure 1. Finally, the strengths and limitations have been restructured for better coherence.

2. *It is my opinion that an initial survey of an CRCT is exploratory and does not need a separate hypothesis (especially not a post hoc one). Further, the post hoc hypothesis added in this revision is ineffectual since it is not related to the comparison between the groups.*

Thank you for your valuable feedback. However, we would like to clarify that while the baseline survey for the CRCT was used to inform the intervention, it provides important data that reflects the ground reality and we think it should be shared. We also wish to highlight that participant allocation to the intervention and the comparison arms was only done after the completion of the baseline survey. Therefore, for this CRCT, the baseline survey functions as a cross-sectional survey rather than just an exploratory one.

3. *The methodology section mentions chi square, t-test and ANOVA. These are parametric test and assume normality. Has the distribution been checked? Since the authors mention most students belong to the lower SES the results may also be skewed.*

We thank the reviewer for providing us with the opportunity to clarify this important aspect of the manuscript. Yes, the distribution were checked before carrying out the analysis. We would like to clarify that as the study is carried out in the public schools, we mentioned they mostly cater to the lower socio economic strata of the population not that the most adolescents were from lower SES. We had checked the normality of socio-economic status (SES) of the participants before carrying out the formal analysis, which was normally distributed as well.

4. *There are some findings that seem inconsistent. The discussion mentions that the prevalence of overweight and obesity was 12% among girls and 9% in boys (Line 278), whereas Figure 3 has overweight at 27 and 22% respectively. Further according to Figure 2 only 13% and 6% of girls and boys respectively have adequate energy intake which is not consistent with finding of overweight and obesity. The age distribution mentioned 10-16 is quite broad for a single grade. Is this expected and accurate? Does this impact the results?*

We thank the reviewer for the opportunity to clarify these points. In line 278, we provided the proportions of obesity among girls and boys, and we have revised the sentence to improve clarity. Regarding Figure 3, the proportion shown represents the combined overweight and obese categories for both girls and boys.

We acknowledge that in Figure 2, only 13% of girls and 6% of boys had adequate energy intake. However, we would like to clarify that the criteria for assessing energy intake and obesity were different. Energy intake was assessed using 24-hour dietary recalls, whereas overweight and obesity were assessed using objective measures, such as BMI. Additionally, obesity is influenced by multiple factors beyond energy intake, including a high carbohydrate diet (as shown in Figure 2), particularly refined carbs, genetics, and a lack of physical activity.

We also agree that the age distribution was broad, but the mean and median age of the participating adolescents was 13 years, which was expected. We checked the distribution of age categories before the analysis, and it was found to be normally distributed.

5. *The manuscript needs a full revision and copy editing to weed out grammatical and typographical errors. I've mentioned a few examples below*

Thank you. All your suggestions are valuable and have helped us improve our manuscript. As suggested we have conducted a thorough revision and copy-editing process to address grammatical and typographical errors throughout the document.

a. *Please consider the statement " Adolescents were deficient in most macro and micro-nutrients, such as energy, fats, fibre, iron, zinc, iodine, riboflavin, and vitamins B-6 and B-12" within the results section in the abstract. Is energy considered a macronutrient? If not, the sentence should be rephrased.*

Thank you for your valuable feedback. We have revised the statement.

b. *Some sentences have confusing syntax and need to be rephrased: Example line 29 "Owing to expensive, lifelong treatments and loss of productive life years due to the increasing premature deaths and disabilities related to various chronic diseases in India, it is recommended to focus more on promotion and prevention than curative measures."*

Thank you, we have revised the line to make it more clear.

c. *The term "normal abdominal obesity" has been used multiple times in the manuscript. Normal obesity is an oxymoron. please use a more accurate term.*

Thank you for bringing this to our notice. We have used the more accurate term of ‘in-range abdominal obesity.’

d. *Line 292 on Page 14. "The mean WHR of the boys and girls were below the WHR ratio cut-offs of 0.85 and 0.9 respectively....". When using the term "respectively" the two sets of nouns should be in the same order. This sentence implies that the cutoff for boys is 0.85 and for girls 0.9.*

Thank you for getting this to our notice. We have revised the sentence based on the suggestion.

e. *Figure 3 says severe acute malnutrition by MUAC and the cut off in legend is MUAC≥ 23/22 cms. The symbol "≥" connotes "greater than" and is inaccurately used here.*

Thank you. We have made the correction.
